# Manganese(iii) complexes stabilized with N-heterocyclic carbene ligands for alcohol oxidation catalysis†

**DOI:** 10.1039/d3dt01013a

**Published:** 2023-05-17

**Authors:** Giacomo Rigoni, Pamela V. S. Nylund, Martin Albrecht

**Affiliations:** a Department of Chemistry, Biochemistry and Pharmaceutical Sciences, University of Bern Freiestrasse 3 CH-3012 Bern Switzerland martin.albrecht@unibe.ch

## Abstract

The chemistry of N-heterocyclic carbenes with Earth-abundant manganese has largely focused on low-valent systems for reductive catalysis. Here, we have decorated imidazole- and triazole-derived carbenes with phenol substituents to access higher-valent Mn(iii) complexes [Mn(*O*,*C*,*O*)(acac)], where acac = acetylacetonato, and *O*,*C*,*O* = bis(phenolate)imidazolylidene (1) or bis(phenolate)triazolylidene (2). Both complexes catalyze the oxidation of alcohols in the presence of *t*BuOOH as terminal oxidant. Complex 2 is slightly more active than 1 (TOF up to 540 h^−1^*vs.* 500 h^−1^), yet significantly more robust towards deactivation. Secondary and primary alcohols are oxidized, the latter with high selectivity and essentially no overoxidation of the aldehyde product to carboxylic acids unless the reaction time is substantially extended. Mechanistic investigations using Hammett parameters, IR spectroscopy, isotope labelling experiments, and specific substrates and oxidants as probes support the formation of a manganese(v) oxo system as the active species and subsequent turnover-limiting hydrogen atom abstraction.

## Abstract 2

Manganese(iii) complexes containing a bis(phenolate)carbene ligand catalyze the oxidation of alcohols and amines using *t*BuOOH as terminal oxidant. Mechanistic investigations indicate a Mn(v) oxo active species that induces hydrogen atom abstraction.

## Graphical abstract

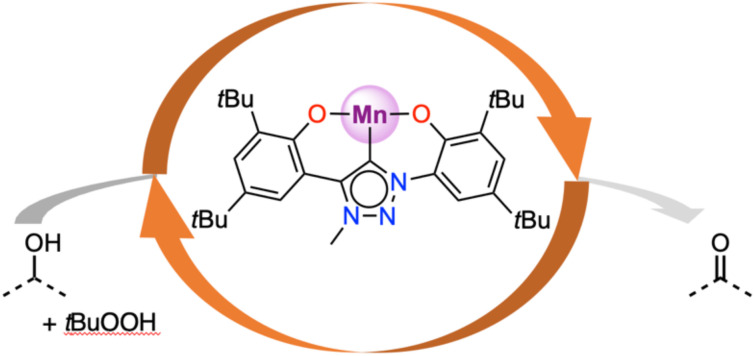

## Introduction

**Fig. 1:**
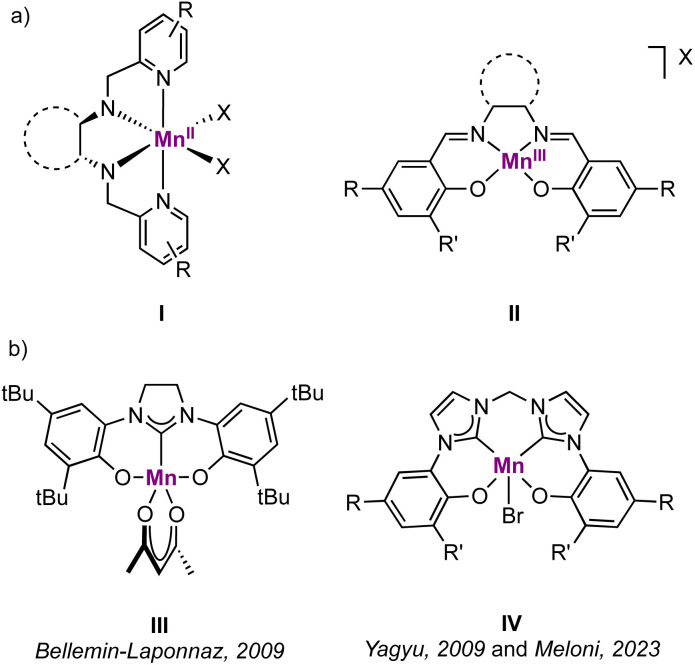
(a) Examples of higher-valent Mn complexes with catalytic activity in oxidation reactions (ref. 9–11); (b) known Mn(iii) complexes containing a NHC ligand (ref. 27–29).

Manganese is one of the most attractive transition metals for homogeneous catalysis, largely due to its high Earth-abundance and biocompatibility, which surpasses most other transition metals.^1^ While it has long been considered to be catalytically only poorly active, recent work demonstrated that manganese-based homogeneous catalysis is viable.^1–3^ Major contributions stem from Mn(i) pincer complexes, which have been used in synthetically relevant reactions, such as reduction, dehydrogenation and dehydrogenative coupling reactions.^4–7^ In contrast, higher-valent Mn(ii) and Mn(iii) compounds have been less widely employed, with most applications related to redox catalysis, *e.g.* C–H hydroxylation.^8^ For those complexes, the ligand coordination environment has been limited by and large to nitrogen and oxygen-based systems^9–11^ (Fig. 1a, **I-II**) with a lack of organometallic species, for instance involving carbene ligands. Indeed, while several examples of manganese(i) N-heterocyclic carbene (NHC) complexes are known,^12–18^ the NHC coordination chemistry has been investigated only scarcely with Mn in higher oxidation states. Especially manganese(iii) NHC complexes are very rare, possibly due to the mismatch between the hard metal center and the soft carbene ligand.^19^ Even with potentially harder mesoionic carbenes (MICs) as a subclass of NHCs,^20–22^ only a single example of a Mn(iii) complex has been reported so far.^23^ This underrepresentation is remarkable, when considering the strong σ-donor character of NHCs in general and of MICs in particular, and their beneficial effects in a variety of catalytic oxidation reactions.^24–26^

The first manganese(iii) complex with an Arduengo-type NHC ligand was reported in 2009 by Bellemin-Laponnaz *et al.* and features an imidazolinylidene as central carbene ligand stabilized with two chelating phenolate groups (Fig. 1b, III).^27^ No catalytic applications of this complex were reported. Yagyu *et al.* reported a related Mn(iii) complex containing a *O*,*C*,*C*,*O*-tetradentate bis(carbene)bis(phenolate) ligand as a mimic of the well-known salen environment that was active in styrene epoxidation by using PhIO as oxidant (Fig. 1b, IV).^28^ This system was very recently applied also as catalyst for the cycloaddition reaction of epoxides with CO_2_.^29^ A few examples of (nitrido) Mn(iv) and Mn(v) complexes with Arduengo-type NHC ligands have been isolated,^30–32^ which undergo several redox processes without any decomposition, including one-electron reduction to the Mn(iii) NHC species. The stability of these complexes may be attributed to the large steric shielding around the high-valent metal center, a configuration that also suggests limited potential for catalytic applications.

Here we have expanded the range of high-valent manganese NHC systems by leveraging on the combination of hard phenolates as support for strongly σ-donating carbenes in first-row transition metal complexes.^33–35^ Specifically, we have prepared Arduengo-type NHC and sterically related mesoionic 1,2,3-triazolylidene complexes with a manganese(iii) center and explored their catalytic activity in alcohol oxidation using *t*BuOOH as sacrificial oxidant. This methodology therefore provides an approach for employing Earth-abundant metals in combination with an inexpensive oxidant^36,37^ for this industrially relevant reaction.^38^ Unlike most other Mn-based catalysts,^36,39–41^ our system does not require acid additives to activate the peroxide, a limitation especially when considering large-scale applications. The new complexes reach appreciable activity with turnover frequencies up to 540 h^−1^ and allow for preliminary mechanistic insights.

## Results and discussion

### Synthesis of ligands and complexes

**Scheme 1:**
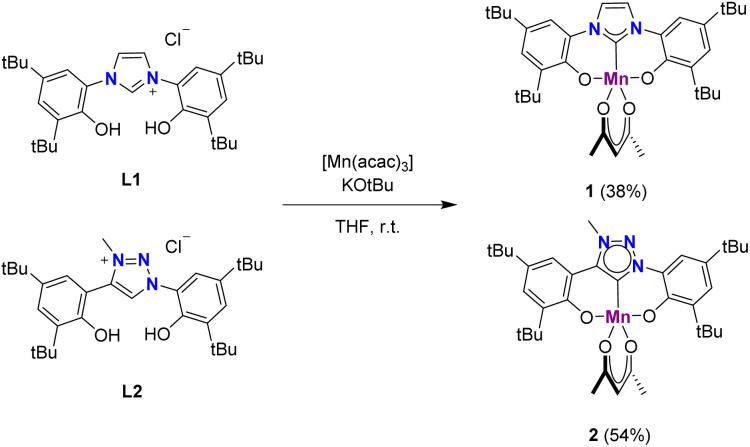
Synthetic procedure for complexes 1 and 2.

Ligands L1 and L2 were synthesized according to literature procedures^42,43^ and metalated with [Mn(acac)_3_] in the presence of KO*t*Bu to afford complexes 1 and 2 in 38% and 54% yield, respectively, as brown powders (Scheme 1). This procedure has been slightly adapted from a previously established method for the synthesis of manganese complexes from imidazolinium salts.^27^ Notably, complex 2 is the second 1,2,3-triazolylidene complex containing a Mn(iii) center^23^ and the first applied in catalysis.

**Fig. 2:**
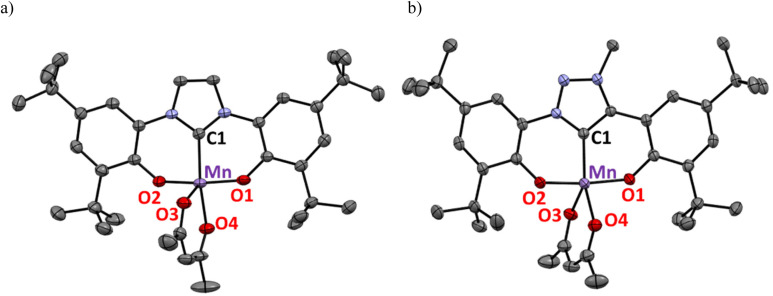
Thermal ellipsoid plots of complex 1 (a) and complex 2 (b; both at 50% probability level, hydrogen atoms omitted for clarity).

The ^1^H NMR spectra of the complexes in CD_3_CN showed the expected paramagnetic character with broad signals in the *δ*_H_ = +25 to −50 ppm range for 1, and +23 to −16 ppm range for 2, respectively (Fig. S1†). While the broadness of the signals prevented an unambiguous assignment, the lower number of signals for 1 compared to that for 2 is consistent with the lower degree of symmetry of the triazolylidene *vs*. imidazolylidene ligand. For both complexes, single crystals suitable for X-ray diffraction analysis were grown upon recrystallization from hot concentrated MeCN solutions (Fig. 2). The molecular structures show a slightly distorted square pyramidal geometry with a *τ*_5_ parameter^44^ of 0.18 for complex 1 and significantly more distorted towards a trigonal bipyramidal geometry for complex 2 (*τ*_5_ = 0.47; Table 1). Both complexes feature a Jahn–Teller distortion, typical for Mn(iii) d^4^ complexes.^27,28^ Specifically, the acac O3 nucleus in pseudo-apical position is more distant to the Mn center than the equatorial O4 (Δ*d* = 0.11 and 0.06 Å for complexes 1 and 2, respectively). The Mn–C bond length in the triazolylidene complex 2 is slightly shorter than in the imidazolylidene analogue (1.955(1) *vs*. 1.979(3) Å), which was attributed to the stronger σ-donor character of the triazolylidene ligand.^20,45^ This bond is also shorter than the Mn–C bond in related Mn(iii)–imidazolylidene complexes^27,28^ and complex III containing an imidazolinylidene ligand (*cf.*Fig. 1, Mn–C >1.98 Å).^27^

**Table 1:** Selected bond lengths (Å) and angles (°) for complex 1 and 2

Complex	1	2
Mn–C1	1.979(3)	1.9550(14)
Mn–O1	1.858(3)	1.8614(10)
Mn–O2	1.853(2)	1.8622(10)
Mn–O3	2.083(3)	2.0522(11)
Mn–O4	1.976(2)	1.9917(11)
O2–Mn–C1	87.3(1)	87.78(5)
O1–Mn–C1	87.0(1)	86.04(5)
O1–Mn–O2	169.49(12)	173.31(5)
C1–Mn–O4	158.56(13)	145.04(4)
*τ*_5_ a	0.18	0.47

a Trigonal distortion parameter (*τ*_5_) calculated according to ref. 44 as *τ*_5_ = (*β* − *α*)/60° with *β* > *α*, where *α* and *β* are the largest valence angles in the five-coordinate species, in this case the O1–Mn–O2 and the C1–Mn–O4 angles.

The electrochemical properties of complexes 1 and 2 were investigated by cyclic voltammetry analysis in MeCN solutions (Fig. S2†). For both complexes, multiple oxidation and reduction events were observed in the 0 to +2.0 V potential range (potentials *vs.* Ag/AgCl), some of which may be quasi-reversible. Notably, coordinating aryloxide moieties of ancillary ligands are known for their redox-noninnocent behavior,^46^ potentially imparting unique redox features to their corresponding metal complexes. Due to the complexity of the curves and the poor investigation of oxidation of NHC–Mn(iii) to Mn(iv) and Mn(v) in literature by cyclic voltammetry, a detailed assignment of the redox events was not possible. Tentatively, the quasi-reversible redox processes occurring at very similar potentials for the two complexes, at *E*_1/2_ = +1.24 V (Δ*E*_p_ = 0.13 V) for 1 and at +1.23 V (Δ*E*_p_ = 0.14 V) for 2 may be attributed to ligand-centered processes.^47,48^ Despite the limited characterization, these electrochemical properties indicate a high potential for these complexes to engage in (catalytic) redox processes.

Complex 1 and 2 are stable for extended times in the solid state and when dissolved in MeCN at 25 °C. In solution at 60 °C, complex 1 starts to decompose after 2 days as indicated by the appearance of resonances due to the free imidazolium salt in the ^1^H NMR spectrum. In contrast, complex 2 did not reveal any visible nor spectral change in MeCN even when kept for one week at 80 °C.

**Fig. 3:**
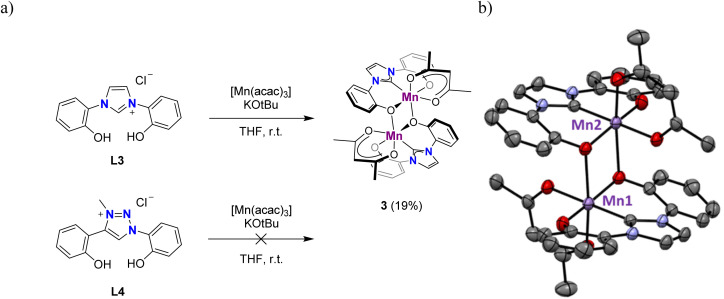
(a) Synthesis of complex 3 from L3 featuring no substituents on the phenolate, while L4 is not reactive; (b) molecular structure of dimetallic complex 3 (thermal ellipsoids at 50% probability level; hydrogen atoms omitted for clarity).

The *t*Bu substituents on the phenolate ligand sites are critical to obtain defined, monometallic complexes. Thus, application of the same metalation procedure to ligand L3, synthesized according to literature procedures,^49^ gave the octahedral dimetallic complex 3 in a low 19% yield (Fig. 3a). The dark brown powder is insoluble in MeCN, MeOH, toluene, or CH_2_Cl_2_, and only sparingly soluble in DMSO. However, crystals obtained from a hot MeCN solution of the crude mixture collected from the reaction with L3 were identified as a dimeric complex, 3, in which two phenolate units from two distinct ligand molecules bridge the two Mn centers (Fig. 3b). Similar dimer formation is presumably prevented in complexes 1 and 2 due to the steric shielding of the metal center by the *t*Bu substituents. Notably, the analogous triazolium salt L4^50^ did not produce any detectable complex when subjected to identical metalation conditions. Instead, the ligand precursor was recovered as the major component of the reaction mixture. Due to its poor solubility, complex 3 was not further investigated.

### Catalytic activity

Complexes 1 and 2 were evaluated as catalysts for the oxidation of secondary alcohols into their corresponding ketones using *t*BuOOH (TBHP) as sacrificial oxidant (Table 2). 1-Phenylethanol was chosen as model substrate. Initially, the reaction was conducted at 80 °C in MeCN using 1.5 eq. of TBHP. Under these conditions, both complexes 1 and 2 displayed good activity with 60% and 70% conversion, respectively, and >95% selectivity towards the ketone after 1 h (entries 1 and 2). However, the reactions did not reach full conversion, and even after extended reaction times (8 h), conversion stalled at 69% and 80%, respectively (Fig. S3a†). Notably, an instantaneous color change from orange to dark brown occurred upon TBHP addition, and after about 90 min, a brown solid started precipitating from the catalytic mixture. At this time, the catalytic activity also drastically decreased. This reduced activity is evident when plotting the variation of substrate concentration on a logarithmic scale, *i.e.* assuming a first-order substrate dependence (Fig. S3b†). In this representation, two catalytic regimes are distinguishable: a first one with high activity in the first 90 min, and a second one with much lower performance after about 2 h.

**Table 2:** Oxidation of 1-phenylethanol with complex 1 and 2^a^

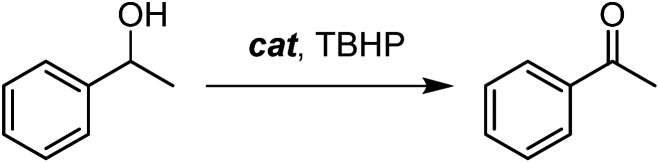						
Entry	Complex	Temperature (°C)	TOF_max_ (h^−1^)	Conversion (yield)^b^ %		
				30 min	1 h	8 h
1	1	80	500	55 (52)	60 (58)	69 (67)
2	2	80	540	62 (60)	70 (67)	80 (79)
3	1	60	280	53 (51)	66 (63)	81 (79)
4	2	60	290	54 (53)	66 (63)	94 (93)
5	2	50	130	35 (33)	54 (53)	89 (86)
6	2	40	97	21 (20)	32 (30)	84 (82)
7	2	25	39	6 (6)	10 (9)	24 (22)
8	Mn(acac)_3_	60	61	17 (16)	23 (21)	69 (65)
9	—	60	—	7 (7)	10 (8)	20 (18)

a Reaction conditions: 0.5 mmol substrate, 0.75 mmol TBHP, 1 mol% Mn complex, 0.06 mmol 1,3,5-trimethoxybenzene, 0.6 mL MeCN, 8 h.

b Determined by GC-FID analysis using 1,3,5-trimethoxybenzene as internal standard.

**Fig. 4:**
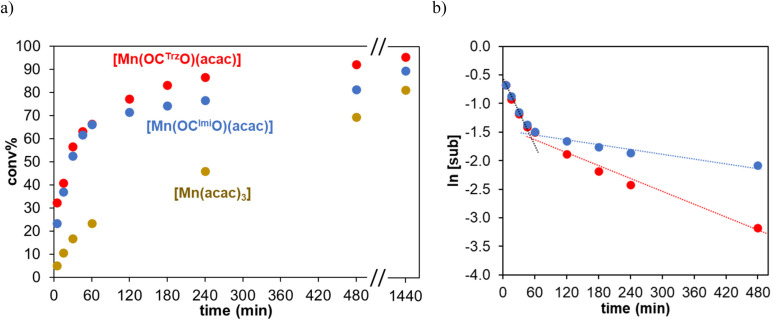
(a) Kinetic profiles of the oxidation of 1-phenylethanol catalyzed by 1 (in blue), 2 (in red) and [Mn(acac)_3_] (in ochre) at 60 °C; (b) logarithmic graph of the profile of 1 (in blue) and 2 (in red). Conditions: 0.5 mmol substrate, 0.75 mmol TBHP, 1 mol% Mn complex, 0.06 mmol 1,3,5-trimethoxybenzene, 0.6 mL MeCN, 60 °C, 24 h.

To probe whether deactivation is a consequence of thermal instability of the catalytically active species, oxidation reactions were carried out at 60 °C. At this reduced temperature, the initial TOF values are, as expected, lower, yet the long-term performances improved considerably (Fig. 4a). Complex 1 reached 81% conversion and 79% yield after 8 h (entry 3), while complex 2 accomplished 94% conversion with essentially full selectivity (93% yield, entry 4). Kinetic analyses indicate that catalyst deactivation is still occurring, though at much slower rate, especially for the triazolylidene complex 2 (Fig. 4b). Therefore, the catalytic performance of complex 2 was analyzed at different reaction temperatures (entries 5–7; Fig. S4†). At 50 and 40 °C, the substrate was converted more slowly, though still >80% substrate oxidation was noted after 8 h (entries 5 and 6). Even at 25 °C, catalytic oxidation took place, though conversion only reached 24% within the same reaction time (entry 7). The beneficial effect of the *O*,*C*,*O*-tridentate triazolylidene ligand, and to a lesser extent also of the imidazolylidene analogue, was shown by using [Mn(acac)_3_] as catalyst precursor. The initial TOF with [Mn(acac)_3_] was five times lower than that of complex 2 (61 *vs.* 290 h^−1^, entry 8 *vs.* 4) and the oxidation stopped at 69% conversion. In the absence of a Mn source, only 20% of the substrate was converted (entry 9), indicating that both the Mn center and the carbene ligand are required for imparting high catalytic activity. It is worth noting that both complex 1 and especially complex 2 show improved activity compared to mononuclear manganese(i) and dinuclear manganese(0) NHC complexes.^17^ While the manganese(0) species accomplished full conversion in 2 h even at slightly lower temperature, complexes 1 and 2 feature catalytic rates that are six-fold higher (TOF_max_ = 500 and 540 *vs.* 90 h^−1^, respectively) and set a new benchmark for alcohol oxidation with NHC manganese catalysts. We note that related penta-coordinate Mn(iii)–salen and -salphen catalysts show considerably higher activity in alcohol oxidation (quantitative conversion in minutes),^9,51–54^ even though using (NBu_4_)HSO_5_ as a harsher terminal oxidant.

Lower conversion values of 70% and 40% were reached when the loading of complex 2 was reduced to 0.5 and 0.1 mol%, respectively (Fig. S5a†), indicating a maximum TON of around 400. The linear correlation between the observed rate constants and the catalyst loading indicates that the reaction is first order in the complex (Fig. S5b†). The relevance of the oxidant was evaluated by changing the amount of TBHP in runs performed at 60 °C. An increase of TBHP from 1.5 to 2.0 eq. induced a slightly higher 97% conversion with consistently high selectivity (Fig. S6a†). When the loading was dropped to 1.0 eq., the conversion lowered to 73% conversion after 8 h. The evaluation of the initial rates suggests no dependence of the rate on the TBHP concentration (Fig. S6b†), which points to a fast and potentially irreversible oxidation of complex 2. Higher concentrations of TBHP >2.5 eq. were detrimental to catalytic activity, presumably due to ensuing overoxidation. Notably, portioned addition of TBHP changed the initial catalytic activity, though it did not improve the final conversion and therefore does not constitute an improvement of the process (Fig. S7†). These results suggest that overoxidation is not a major issue under the selected conditions, nor that significant catalyst deactivation occurs in the presence of a moderate excess of oxidant at the onset of the catalytic reaction. It is noteworthy that other terminal oxidants such as air, aqueous 30% H_2_O_2_, or aqueous 10% NaOCl did not produce any significant quantities of ketone in the presence of catalytic amounts of complex 2, suggesting that TBHP plays a molecular role in the catalytic process.

A selection of organic solvents was screened for the oxidation reaction, though MeCN provided the best results (Table 3 and Fig. S8†). A potential correlation with polarity was probed by using apolar toluene and slightly more polar THF, as well as highly polar DMF (entries 2–4),^55^ though no trend was detected and the performance of complex 2 in these solvents was consistently poor. Likewise, DMSO had a negative effect, which might be due to its metal coordination ability and ensuing competition with coordination of the oxidant or the substrate (entry 5). No dimethylsulfone was observed that would originate from solvent oxidation. Reactions in 2,2,2-trifluoroethanol (TFE) were tested because this solvent has been employed previously for efficient metal-based oxidation reactions.^56–58^ However, only 68% conversion was reached after 8 h, considerably less than in MeCN yet respectable when compared to other solvents (entry 6). 1,2-Dichloroethane (DCE), a higher-boiling point analogue of CH_2_Cl_2_, gave an appreciable 75% conversion.

**Table 3:** Oxidation of 1-phenylethanol with complex 2 in different solvents^a^

Entry	Solvent	% conversion^b^
1	MeCN	94
2	THF	35
3	Toluene	39
4	DMF	28
5	DMSO	24
6	CF_3_CH_2_OH (TFE)	68
7	ClCH_2_CH_2_Cl (DCE)	75

a Reaction conditions: 0.5 mmol substrate, 0.75 mmol TBHP, 1 mol% complex 2, 0.06 mmol 1,3,5-trimethoxybenzene, 0.6 mL solvent, 60 °C, 8 h.

b Determined by GC-FID analysis using 1,3,5-trimethoxybenzene as internal standard.

**Fig. 5:**
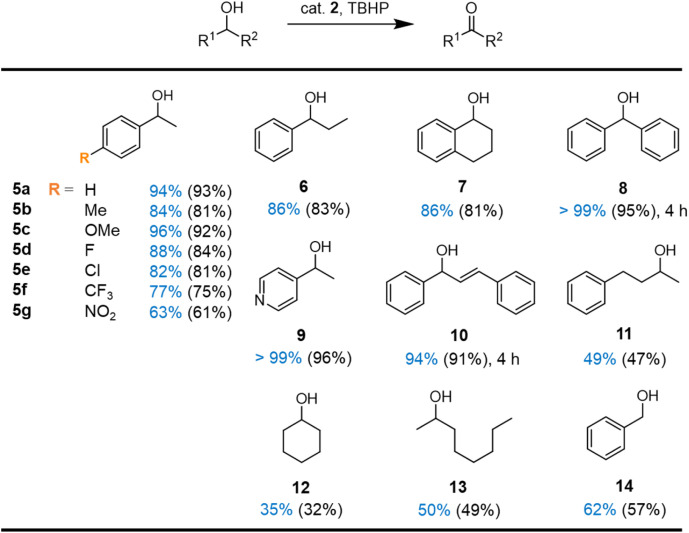
Scope of the oxidation reaction with complex 2. Reaction conditions: 0.5 mmol substrate, 0.75 mmol TBHP, 1 mol% complex 2, 0.06 mmol 1,3,5-trimethoxybenzene, 0.6 mL MeCN, 60 °C, 8 h. Conversions (in blue) and yields (in parenthesis) were determined by ^1^H NMR spectroscopy using 1,3,5-trimethoxybenzene as internal standard.

Based on these optimized reaction conditions for complex 2, a small substrate scope was performed. Complex 2 was effective in the catalytic oxidation of a wide variety of secondary alcohols (Fig. 5). Both electron-donating and -withdrawing substituents (substrates 5b–g) on the aromatic ring of 1-phenylethanol were well-tolerated with high selectivity towards ketone formation. However, the reaction is significantly slowed down with electron-deficient systems. For example, *para*-nitro substitution reduced the initial turnover frequency from 290 h^−1^ for the unsubstituted system to 100 h^−1^, and consequentially, the conversion after 8 h reached only 63% as opposed to the 94% with the parent substrate (substrates 5a*vs.*5g, *vide infra*).

Increased steric bulkiness on the side of the aliphatic chain does not affect the catalytic performances (substrates 6 and 7). Not surprisingly, oxidation of diphenylmethanol 8 reached completion much faster (4 *vs.* 8 h), since the benzylic position is doubly activated. Similarly, the pyridyl-substituted alcohol 9 is converted fully, pointing towards a good tolerance of heterocycles. Oxidation of the activated allylic alcohol 10 gave 94% conversion after 4 h with high selectivity towards the enone; no epoxidation products were detected. Even aliphatic alcohols such as 11–13 are converted selectively, though yields reached only around 35–50%. Notably, oxidation of the primary benzyl alcohol 14 gave 62% conversion with excellent selectively within 8 h. Traces of benzoic acid due to overoxidation appeared only after 24 h, (Fig. S29†) suggesting attractive opportunities for the selective oxidation of alcohols to aldehydes with this catalytic system.

**Scheme 2:**
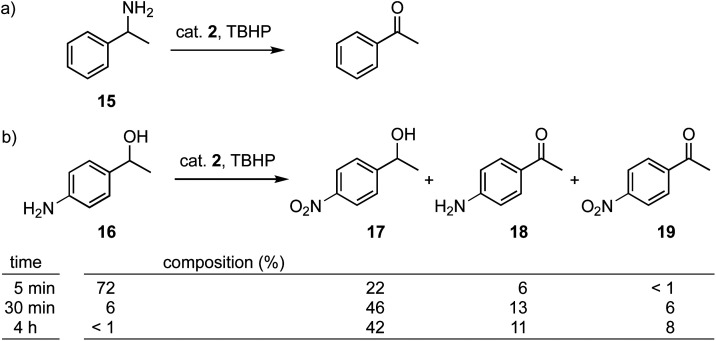
Amine oxidation reaction with complex 2 using (a) 1-phenylethylamine and (b) 4′-amino-1-phenylethanol as substrate. Reaction conditions: 0.5 mmol substrate, 0.75 mmol TBHP, 1 mol% complex 2, 0.06 mmol 1,3,5-trimethoxybenzene, 0.6 mL MeCN, 60 °C, 8 h. The reaction mixture composition was determined by ^1^H NMR spectroscopy using 1,3,5-trimethoxybenzene as internal standard.

In addition to alcohol oxidation, also amine oxidation was probed with complex 2 as catalyst precursor. Under the conditions used for alcohol oxidation, 1-phenylethylamine 15 is converted very rapidly (75% conversion in the first 5 min) and much faster than alcohol oxidation (TOF_max_ = 900 *vs.* 540 h^−1^ for substrate 5a, Fig. S30†), to afford a metastable intermediate that is gradually transformed to acetophenone as the final product (Scheme 2a). While it is tempting to propose the formed intermediate as the corresponding imine, the NMR data do not match with published values.^59^ In contrast, amines without α hydrogens such as anilines are oxidized to nitro functionalities. This oxidation is preferred over alcohol oxidation, as demonstrated with the amino-substituted phenylethylalcohol 16 as a bifunctional substrate. Catalysis with complex 2 afforded a mixture of the nitro-alcohol 17 from amine oxidation, the amino-ketone 18 from alcohol oxidation, and small quantities of the nitro-ketone 19 from oxidation of both functional groups (Scheme 2b). The rate of amine *vs.* alcohol oxidation remains almost constant at about a 3.5 : 1 ratio in favor of amine oxidation, suggesting a competitive pathway with a *ca.* 3 kJ mol^−1^ lower transition state for amine oxidation (Fig. S31†). However, the relatively low yields with respect to conversion (*e.g.* 94% conversion after 30 min, though only a combined yield of 65%) suggests side reactions, which may involve Schiff base reaction of the amine with the ketone and potential oligomerization of the amino-ketone 18. In line with such a reaction trajectory, oxidation of (±)-norephedrine as an aliphatic 1,2-aminoalcohol yielded a complex mixture of products.

### Mechanistic investigations

**Fig. 6:**
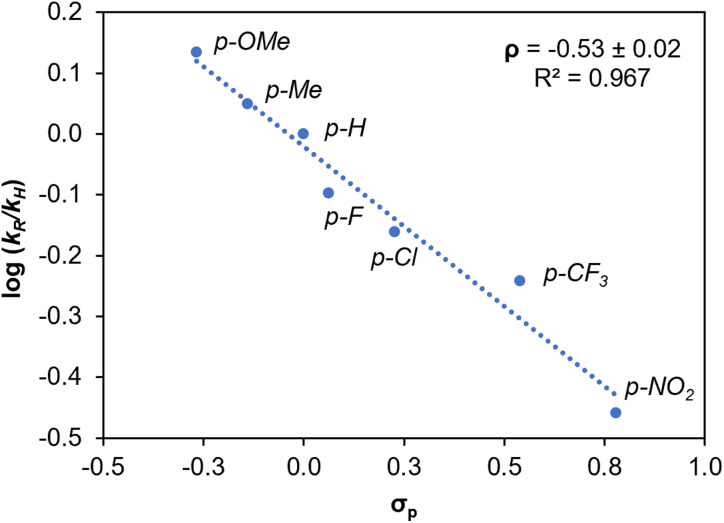
Hammett plot of log(*k*_R_/*k*_H_) *vs.* Hammett parameter (*σ*_p_) for the catalytic oxidation of *para*-substituted 1-phenylethanols. Conditions: 0.5 mmol substrate, 0.75 mmol TBHP, 1 mol% catalyst 2, 0.06 mmol 1,3,5-trimethoxybenzene, 0.6 mL MeCN, 60 °C, 8 h.

To get some insight into the oxidation mechanism, detailed kinetic measurements were carried out. The effect of the electron density distribution in the substrate aryl ring was assessed by comparing the initial rates for the oxidation of a series of *para*-substituted 1-phenylethanols 5a–5g (*cf.*Fig. 5). When compared to the unsubstituted analogue, oxidation is faster with electron-donating groups (negative *σ*_p_ values) and slower with electron-withdrawing groups (positive *σ*_p_ values). A plot of the relative initial rate constants log(*k*_R_/*k*_H_) against the Hammett values *σ*_p_ revealed a linear correlation with a slope *ρ* = –0.53 (Fig. 6). The negative slope indicates the build-up of (partial) positive charge in the transition state that corresponds to the turnover-limiting step, which can be rationalized by the polarization of the C–H bond towards hydrogen atom or hydride transfer. Such a model is consistent with the expected mechanism involving cleavage of the benzylic C–H bond as rate-limiting step of the reaction.^36^ The small value *ρ* of the slope is in line with previous examples in literature.^36,39^ It does not support the formation of a localized cation and thus disfavors a potential transition state comprised of a carbocation, instead suggesting a H˙ abstraction *via* a radical pathway.^60,61^

The relevance of the C–H cleavage step was demonstrated by carrying out the same reaction using 5a–*d* as substrate, *i.e.* 1-phenylethanol that is monodeuterated at the benzylic position and was obtained by reduction of acetophenone with NaBD_4_ (Fig. S9a†). The observed initial rates for the two substrates, determined by independent catalytic runs, were *k*_obs_,_H_ = 3.3 × 10^−4^ s^−1^ for the protic substrate 5a, and *k*_obs,D_ = 4.7 × 10^−5^ s^−1^ for the deuterated isotope 5a–*d* (Fig. S9b†). These rates yield a primary kinetic isotope effect, KIE = 7.0. Such a strong isotope effect indicates that the H-abstracting agent is a metal-oxo or -oxyl species, since free-radical mechanisms usually produce much lower KIEs.^62^ Likewise, Mn(ii)-catalyzed oxidation reactions typically feature lower values (KIE ∼ 2).^36,39^ A similarly strong effect as noted for complex 2 was observed in catalytic oxidations with Mn(iv)-hydroxo species (KIE up to 10),^63,64^ and with metal-alkylperoxo (M-OOR) systems.^38,62^

**Scheme 3:**
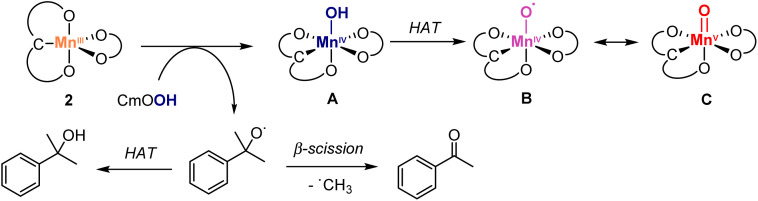
Plausible activation pathways of pre-catalyst 2 with hydroperoxides, featuring sequential homolytic cleavage of O–O and O–H bonds to produce the catalytically active Mn(iv)–O˙ (B) or Mn(v)=O (C; upper section) along with CmO˙ radical, which undergoes spontaneous β-scission to give acetophenone or assists the HAT from A to yield cumyl alcohol (bottom section; both products detected by NMR spectroscopy, Fig. S9†).

To better understand the role of the oxidant, a catalytic run was performed with cumene hydroperoxide (CmOOH) instead of TBHP (Scheme 3). Thus, oxidation of diphenylmethanol 8 with complex 2 and CmOOH under otherwise identical conditions gave benzophenone at considerably lower rates compared to TBHP (61% yield after 8 h *vs*. >95% yield after 4 h, *cf.*Fig. 5). Moreover, the reaction revealed the build-up of acetophenone and cumyl alcohol already after 5 min, identified by GC analysis and the characteristic ^1^H NMR signals at 2.61 and 1.57 ppm, respectively. Time-dependent monitoring of the reaction demonstrated a gradual increase of acetophenone to a 22% yield after 8 h (Fig. S10†). Acetophenone originates in this reaction from homolytic O–O bond cleavage of CmOOH, since the formed CmO˙ alkoxyl radical is known to easily undergo β-C–C bond scission to afford acetophenone and a methyl radical (Scheme 3).^65,66^ Therefore, these experiments support the formation of a Mn-oxo (C) or -oxyl species (B) as catalytically active species, either generated from HO˙ radicals from homolytic ROOH cleavage, or from a transient Mn–OOR species which undergoes rapid O–O bond scission and release of RO˙.^66^

**Fig. 7:**
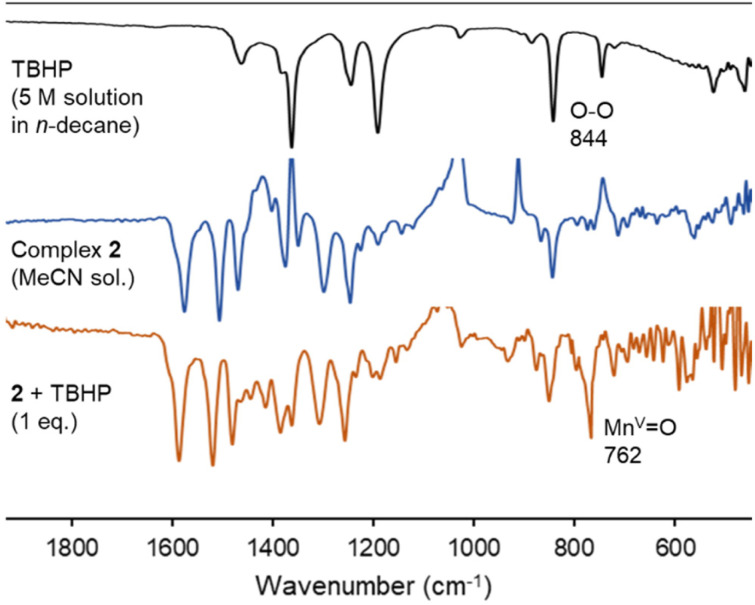
Monitoring of the reactivity of complex 2 upon addition of 1 eq. of TBHP by FT-IR spectroscopy to form intermediate C. Conditions: 10 mM solution of complex 2 in MeCN, 1 eq. TBHP, rt.

The Mn–O species involved in the catalytic cycle was spectroscopically characterized from a MeCN solution of complex 2, which was treated with 1 eq. of TBHP at room temperature. The sample changed color from orange to brown within 5 min, together with the observation of a characteristic absorbance at 350 nm (Fig. S11†). In the IR spectrum, a sharp band at 762 cm^−1^ appeared and was attributed to the Mn(v)=O vibrational stretching (Fig. 7), in good agreement with the data of porphyrin manganese-oxo complexes (*ν*_Mn=O_ = 755–800 cm^−1^).^67,68^ The spectrum was persistent for at least 15 min, indicating some stability of this species C in solution in the absence of substrate. NMR spectroscopic monitoring of the same reaction in CD_3_CN did not show any diagnostic changes in the first 30 min, indicative of the perseverance of a paramagnetic nature of the complex (Fig. S12†). Only at extended reaction times, diamagnetic signals appear in the aromatic region, which were different from those of the triazolium salt L2 and were tentatively attributed to oxidation-derived decomposition products.

**Scheme 4:**
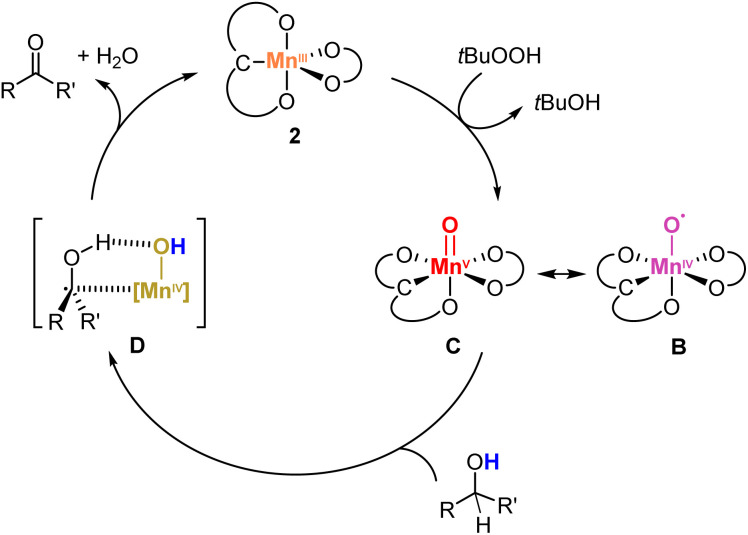
Proposed catalytic cycle for the alcohol oxidation reaction performed by complex 2.

Cyclobutanol oxidation was used as an additional mechanistic probe to distinguish one- and two-electron oxidation processes.^69–71^ Time-dependent monitoring of the reaction by ^1^H NMR spectroscopy over 8 h indicated selective formation of cyclobutanone in 66% yield without any formation of 1-butanal adducts from ring opening (Scheme S1 and Fig. S13†). This reaction outcome suggests a two sequential one-electron redox processes. Specifically, it implies that the adduct D resulting from initial hydrogen atom transfer (HAT) from the substrate by the Mn(v)-oxo species C is sufficiently stable to induce a second HAT involving the RO-H unit of the substrate to form the ketone and H_2_O, also regenerating the Mn(iii) species. A low stability of this adduct would result in the release of the organic radical ensuing ring opening to produce 1-butanal derivatives.^69–71^ Consolidating these mechanistic investigations provides a plausible mechanism for the alcohol oxidation with complex 2 (Scheme 4). Initial oxidation with TBHP yields a Mn(v)=O species C, identified by IR spectroscopy. Subsequent formation of the adduct D*via* HAT from the alcohol substrate is proposed to be turnover-limiting. Such a model is in agreement with the significant KIE, the Hammett correlation observed in the substrate series, and also its negative *ρ* value. A second HAT releases the ketone product and H_2_O from the metal coordination sphere and regenerates the Mn(iii) species 2, thus closing the catalytic cycle.

## Conclusions

We demonstrate here that NHC manganese(iii) complexes are stable despite the unfavorable combination of soft carbene ligands and hard and high-valent Mn(iii) centers. This mismatch has been mitigated by introducing chelating phenolate units on the carbene ligand. The complexes display appreciable catalytic activity in the oxidation of alcohols, thus forming ketones and also aldehydes with remarkably high selectivity and essentially no overoxidation. Mechanistic investigations led to a plausible reactivity involving the formation of a Mn(v)=O species and a rate-limiting hydrogen atom abstraction step. These insights provide opportunities for further improving the catalytic activity. Moreover, they demonstrate the suitability of high-valent Mn NHC systems for oxidation catalysis.

## Experimental section

### General considerations

All reagents and solvents were commercially available and used as received unless specified differently. THF was dried by diffusion through solvent purification columns (SPS). Metalation reactions were carried out under an inert nitrogen atmosphere using standard Schlenk techniques. Ligands L1–4 were synthesized following the reported literature procedures.^42,43,49,50^ NMR spectra were measured at 25 °C on a Bruker spectrometer operating at 300 or 400 MHz (^1^H NMR) and 75 or 101 MHz (^13^C{^1^H} NMR), respectively. Chemical shifts (*δ* in ppm, coupling constants *J* in Hz) were referenced to residual solvent resonances downfield to SiMe_4_. Elemental analyses were performed by the Microanalytic Laboratory of the Department of Chemistry and Biochemistry (DCB) of University of Bern, using a ThermoScientific Flash 2000 CHNS-O elemental analyzer. High-resolution mass spectrometry measurements were carried out using a ThermoScientific LTQ Orbitrap XL (ESI-TOF). GC-FID analysis was performed by using a Agilent 7820A gas chromatograph equipped with a Agilent 19091J-413 HP-5 (30 m × 0.320 mm, 0.25 μm) column. UV-Vis experiments were performed using a Shimadzu UV-1800 UV-Vis spectrophotometer. Infrared spectra were recorded on a JASCO FT/IR-4700 FT-IR spectrometer equipped with ATR PRO ONE single reflection accessory. Cyclic voltammetry measurements were carried out using a Metrohm Autolab Model PGSTAT101 potentiostat employing a gastight three-electrode cell under an argon atmosphere. A platinum disk with 7.0 mm^2^ surface area was used as the working electrode and polished before each measurement. The reference electrode was Ag/AgCl; the counter electrode was Pt foil. Bu_4_NPF_6_ (0.1 M) in dry solvent was used as supporting electrolyte with analyte concentrations of approximately 1 mM. The ferrocenium/ferrocene (Fc^+^/Fc) redox couple was used as an internal reference (*E*_1/2_ = 0.43 V *vs.* SSCE).

### General synthetic procedure of the complexes

In an oven-dried Schlenk flask, the solid ligand LH_3_Cl (1.99 mmol, 1.0 eq.), [Mn(acac)]_3_ (700.0 mg, 1.99 mmol, 1.0 eq.) and KO*t*Bu (334.5 mg, 2.98 mmol, 1.5 eq.) were mixed together under inert nitrogen atmosphere. The solids were suspended in dry THF (15 mL) and the resulting mixture was left stirring at room temperature for 12 h. The suspension was then filtered through a Celite pad and the filtrate was dried under reduced pressure. The crude was recrystallized upon dissolution in hot MeCN for 2 h, affording the corresponding [Mn(L)acac] complex.

### Complex 1

According to the general method, 1021 mg of ligand L1 were used. Complex 1 was afforded in 38% yield (476 mg) as a pale brown powder.

^1^H-NMR (300 MHz, CD_3_CN): *δ* 25.1, 16.2, 2.2, −8.0, −49.3 ppm. ESI-HRMS (MeCN): calcd for C_36_H_50_MnN_2_O_4_ [M + H]^+^, *m*/*z* 629.3151, found *m*/*z* 629.3146. Elemental analysis calcd (%) for C_36_H_49_MnN_2_O_4_·H_2_O (646.318 u): C 66.86, H 7.95, N 4.33; found: C 66.52, H 7.93, N 4.51.

### Complex 2

According to the general method, 1051 mg of ligand L2 were used. Complex 2 was afforded in 54% yield (692 mg) as dark brown crystals.

^1^H-NMR (300 MHz, CD_3_CN): *δ* 22.7, 21.3, 17.3, 2.0, −5.4, −10.3, −16.0 ppm. ESI-HRMS (MeCN): calcd for C_31_H_43_MnN_3_O_2_Na [M − acac + Na]^+^, *m*/*z* 567.2633, found *m*/*z* 567.2637. Elemental analysis calcd (%) for C_36_H_50_MnN_3_O_4_ (643.318 u): C 67.17, H 7.83, N 6.53; found: C 67.16, H 7.90, N 6.75.

### General catalytic procedure

In a 5 mL vial, the catalyst (1 mol%) was dissolved in 0.6 mL of a 0.1 M stock solution of the internal standard, 1,3,5-trimethoxybenzene, in the desired solvent. Then, the substrate (0.5 mmol) was added, the flask was sealed and the resulting mixture stirred and pre-heated at the desired temperature for 5 min. Afterwards, TBHP (1.5 eq., 0.75 mmol, 150 μL of a commercial 5 M solution in *n*-decane) was injected into the mixture. The reaction was monitored by taking aliquots of the catalytic mixture, which were rapidly plunged into an ice bath to quench the reaction, the volume filtered through a neutral Al_2_O_3_ layer and analysed either with GC-FID (1 : 100 dilution in MeOH) or ^1^H NMR spectroscopy in CDCl_3_ according to the initial amount of internal standard added. At the end of the catalysis, products were extracted in *n*-pentane from the MeCN mixture, dried under reduced pressure and identified by comparison between their ^1^H NMR spectra in CDCl_3_ and the literature data.

## Conflicts of interest

The authors declare no competing financial interests.

## Supplementary Material

DT-052-D3DT01013A-s001

DT-052-D3DT01013A-s002
