# Supplementary material for: Manganese(iii) complexes stabilized with N-heterocyclic carbene ligands for alcohol oxidation catalysis
Source: Dalton Trans. 2023 May 17;52(23):7992–8002. doi: 10.1039/d3dt01013a (PMC10262172; doi:10.1039/d3dt01013a)
Supplement: DT-052-D3DT01013A-s001 [file DT-052-D3DT01013A-s001.pdf]

Electronic Supplementary Material (ESI) for Dalton Transactions.
This journal is © The Royal Society of Chemistry 2023

Electronic Supplementary Information

belonging to

Manganese(III) complexes stabilized with N-heterocyclic carbene ligands for alcohol oxidation catalysis

Giacomo Rigoni, Pamela V. S. Nylund and Martin Albrecht*

Department of Chemistry, Biochemistry and Pharmaceutical Sciences, University of Bern, Freiestrasse 3, CH-3012 Bern, Switzerland

Email: martin.albrecht@unibe.ch

Table of contents

1. NMR and CV characterization of the Mn complexes 2
2. Catalytic experiments 4
3. Characterization of the purified oxidation products 11
4. Crystallographic details 22
5. References ....**Error! Bookmark not defined.**

1. NMR and CV characterization of the Mn complexes

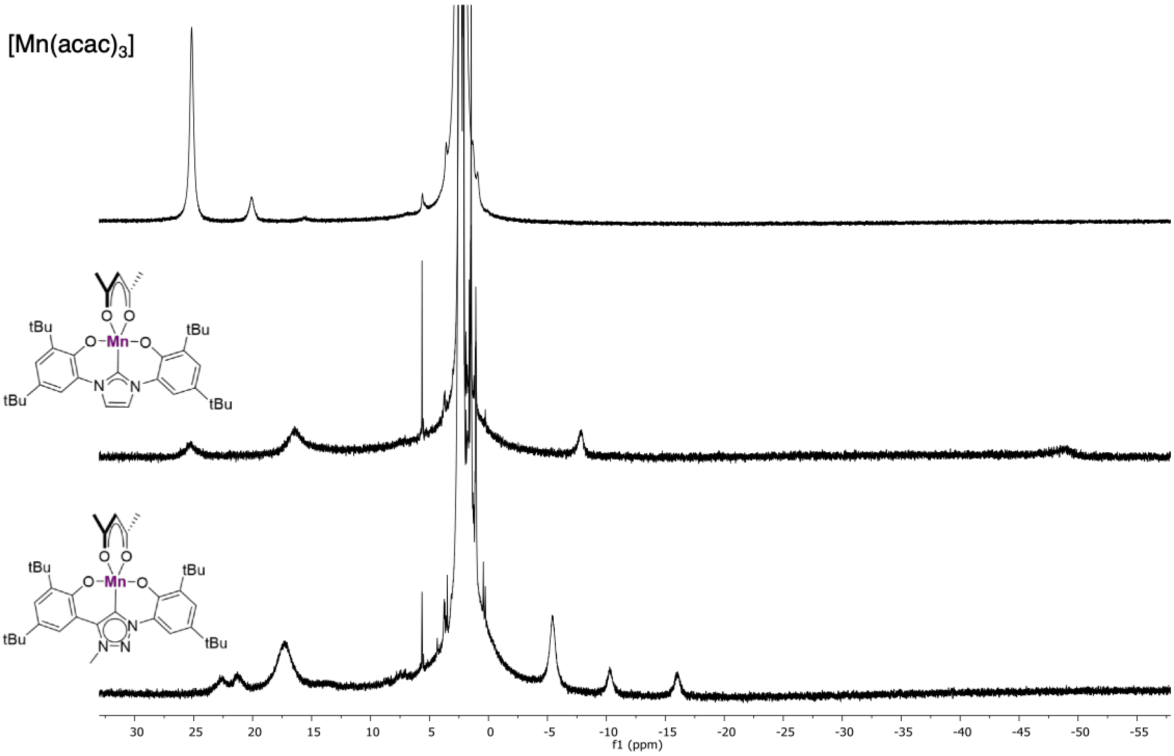

Figure S1. Stacked ^{1}H NMR spectra (CD_3CN, 298 K, 300MHz) of [Mn(acac)$_3$] (top), complex **1** (middle) and **2** (bottom).

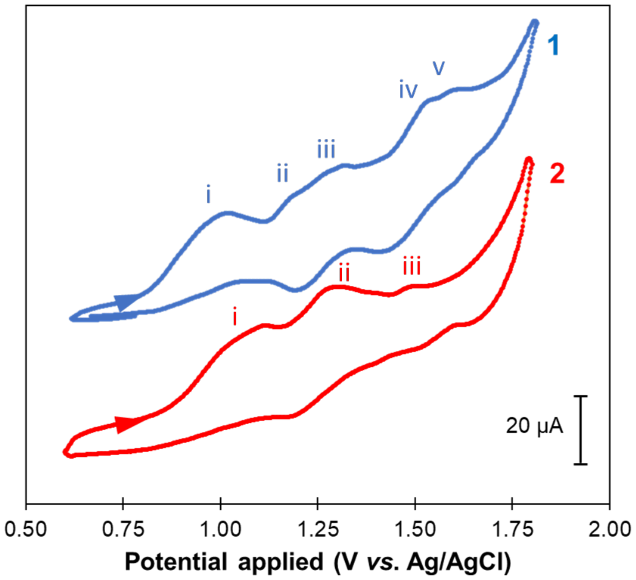

Figure S2. Cyclic voltammograms of Mn(III) complexes **1** (in blue) and **2** (in red). Conditions: 1 mM in MeCN with 0.1 M $TBAPF_6$ as supporting electrolyte, scan rate 500 mVs^{-1}, Fc^+/Fc as internal standard with $E_{1/2}$ = 0.43 V *vs.* Ag/AgCl.

Table S1. Redox potential values of the redox events observed for Mn(III) complexes **1** and **2**.[a)]

process	E_{pa}	E_{pc}	$E_{1/2}$ (ΔE)
**1(i)**	1.005	-	-
**1(ii)**	1.170	-	-
**1(iii)**	1.305	1.178	1.241 (0.127) [b)]
**1(iv)**	1.557	1.391	1.474 (0.166)
**1(v)**	1.596	-	-
**2(i)**	1.115	-	-
**2(ii)**	1.303	1.159	1.231 (0.144) [b)]
**2(iii)**	1.430	-	-

[a)] All values are referenced to Fc^+/Fc as internal standard with $E_{1/2}$ = 0.43 V *vs.* Ag/AgCl.
[b)] The events at $E_{1/2}$ = +1.24 (complex **1**) and $E_{1/2}$ = +1.23 (complex **2**) may tentatively attributed to ligand-centered processes; for metal-centered processes, a lower oxidation potential would be expected for the triazolylidene complex **2** as a consequence of the stronger ligand σ-donation (see refs 20–22 of the main manuscript).

2. Catalytic and mechanistic experiments

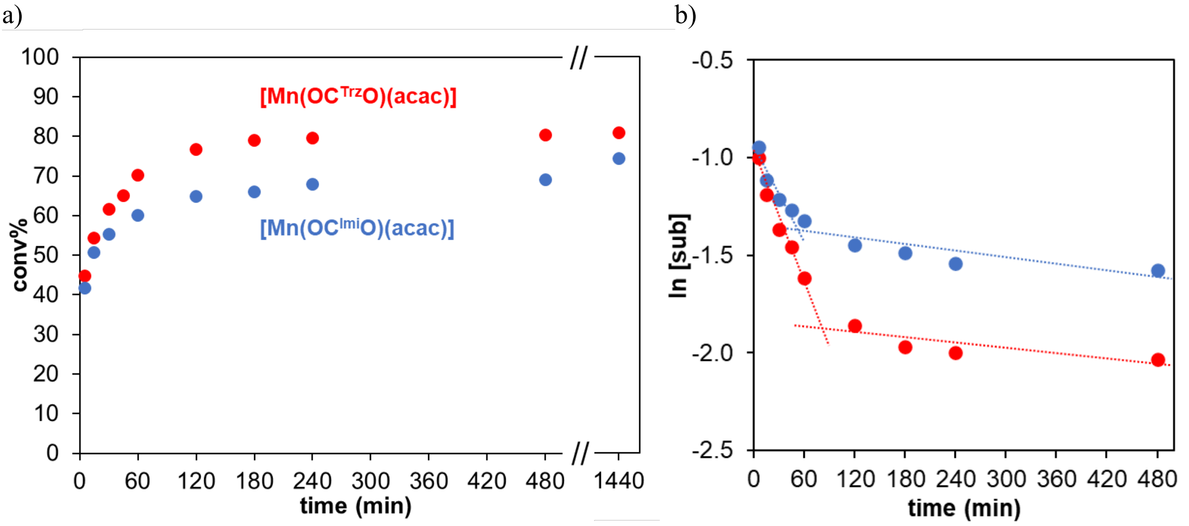

Figure S3. a) Kinetic profiles of 1-phenylethanol oxidation catalyzed by **1** (in blue) and **2** (in red) at 80 °C; b) logarithmic graph of the profile of **1** (in blue) and **2** (in red). Conditions: 0.5 mmol substrate, 0.75 mmol TBHP, 1 mol% catalyst, 0.06 mmol 1,3,5-trimethoxybenzene, 0.6 mL MeCN, 80 °C, 24 h.

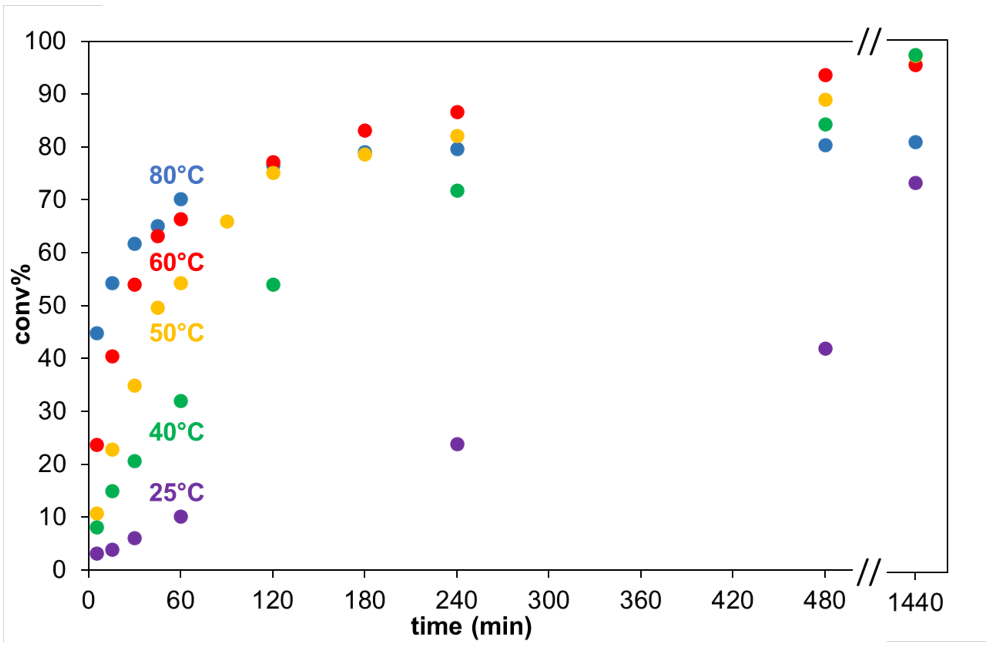

Figure S4. Effect of the temperature on 1-phenylethanol oxidation. Conditions: 0.5 mmol substrate, 0.75 mmol TBHP, 1 mol% catalyst **2**, 0.06 mmol 1,3,5-trimethoxybenzene, 0.6 mL MeCN, 24 h.

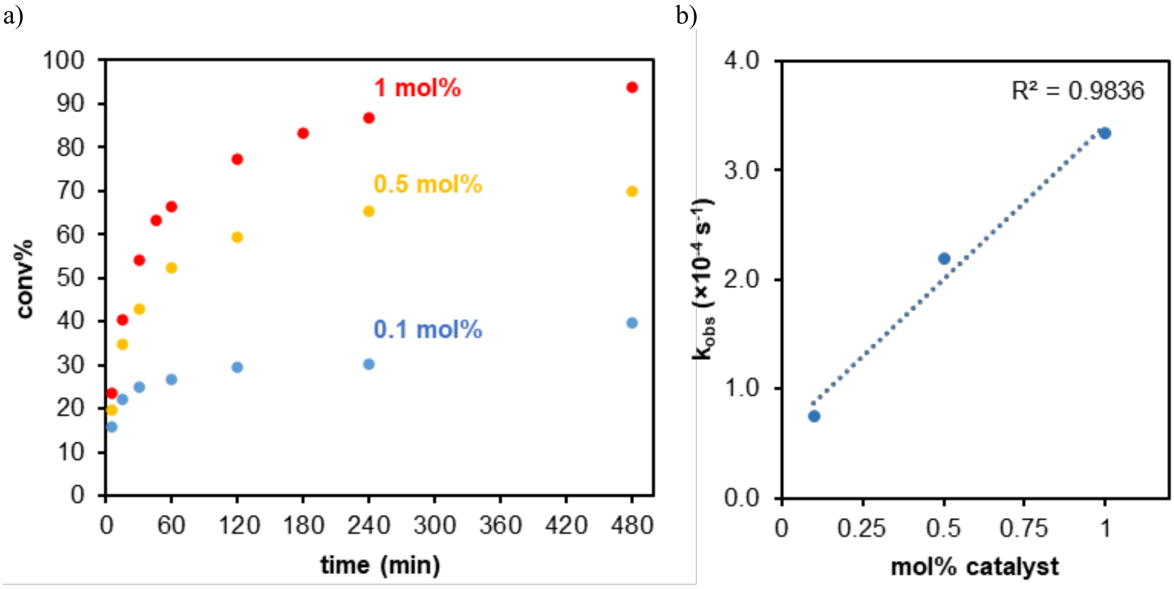

Figure S5. a) Effect of the catalyst loading of **2** on 1-phenylethanol oxidation: 1 mol% (in red), 0.5 mol% (in yellow) and 0.1 mol% (in blue); b) reaction rate *vs.* catalyst loading plot, indicating that the reaction is first order in catalyst. Conditions: 0.5 mmol substrate, 0.75 mmol TBHP, 0.06 mmol 1,3,5-trimethoxybenzene, 0.6 mL MeCN, 60 °C, 8 h.

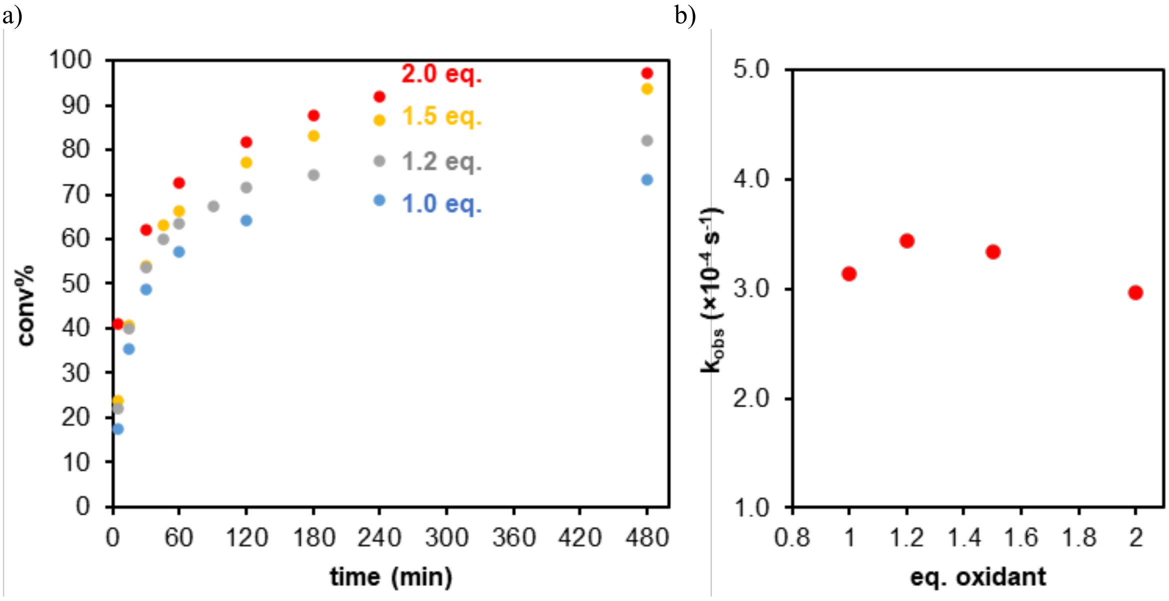

Figure S6. a) Effect of the amount of TBHP on 1-phenylethanol oxidation: 2.0 eq. (in red), 1.5 eq. (in yellow), 1.2 eq. (in grey) and 1.0 eq. (in blue); b) reaction rate *vs.* equivalents of oxidant plot, indicating that the substrate conversion is (initially) independent from the amount of oxidant added. Conditions: 0.5 mmol substrate, 1 mol% catalyst **2**, 0.06 mmol 1,3,5-trimethoxybenzene, 0.6 mL MeCN, 60 °C, 8 h.

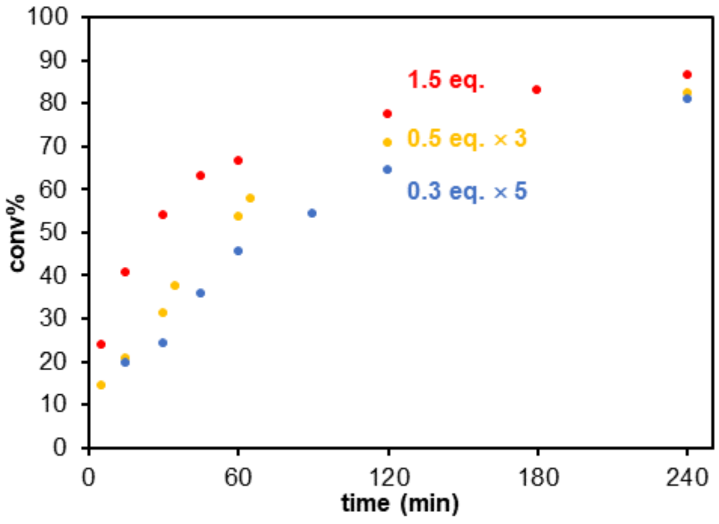

Figure S7. Effect of the addition mode of TBHP on 1-phenylethanol oxidation: single injection at t = 0 (in red), three 0.5 eq. injections at t = 0, 30 and 60 min (in yellow) and five 0.3 eq. injections at t = 0, 30, 60, 90, 120 min (in blue). Conditions: 0.5 mmol substrate, 0.75 mmol TBHP, 1 mol% catalyst **2**, 0.06 mmol 1,3,5-trimethoxybenzene, 0.6 mL MeCN, 60 °C, 4 h.

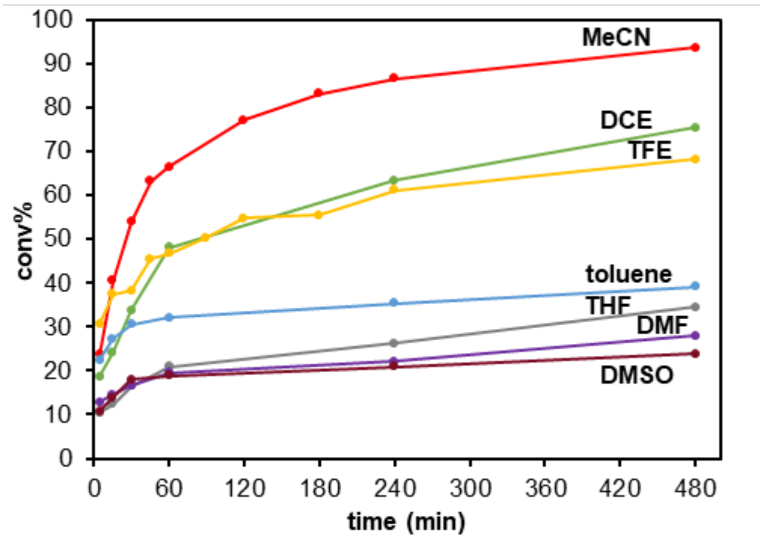

Figure S8. Effect of the solvent on 1-phenylethanol oxidation. Conditions: 0.5 mmol substrate, 0.75 mmol TBHP, 1 mol% catalyst **2**, 0.06 mmol 1,3,5-trimethoxybenzene, 0.6 mL solvent, 60 °C, 8 h.

a)

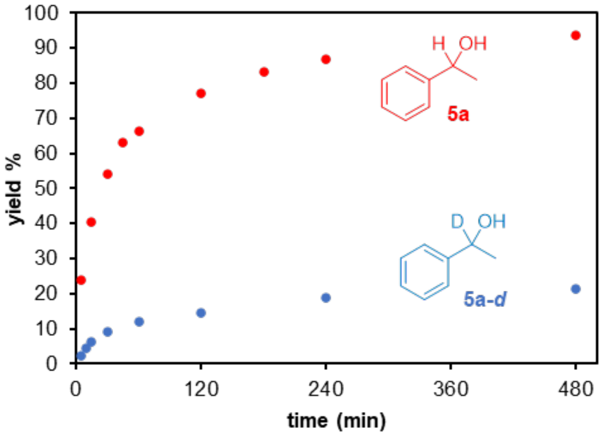

b)

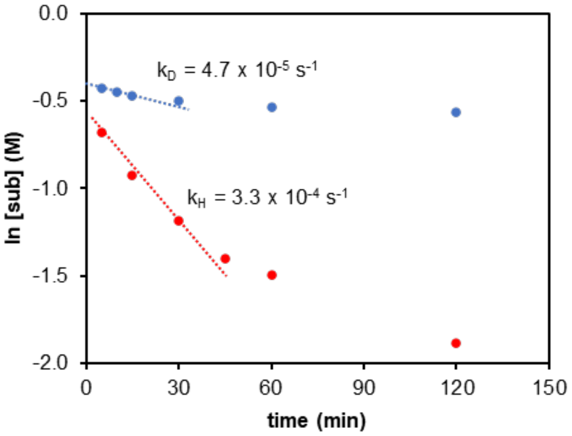

Figure S9. a) Kinetic profiles of the oxidation of 1-phenylethanol (in red) and 1-deuterio-1-phenylethanol (in blue) catalyzed by **2**; b) logarithmic graph of the profile of 1-phenylethanol (in red) and 1-deuterio-1-phenylethanol (in blue). Conditions: 0.5 mmol substrate, 0.75 mmol TBHP, 1 mol% catalyst **2**, 0.06 mmol 1,3,5-trimethoxybenzene, 0.6 mL MeCN, 60 °C, 8 h.

a)

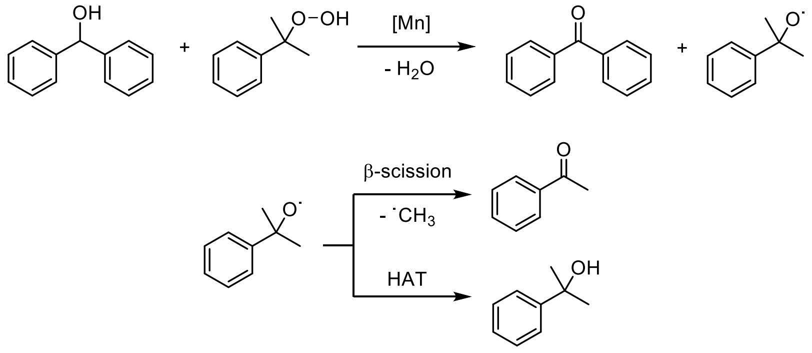

b)

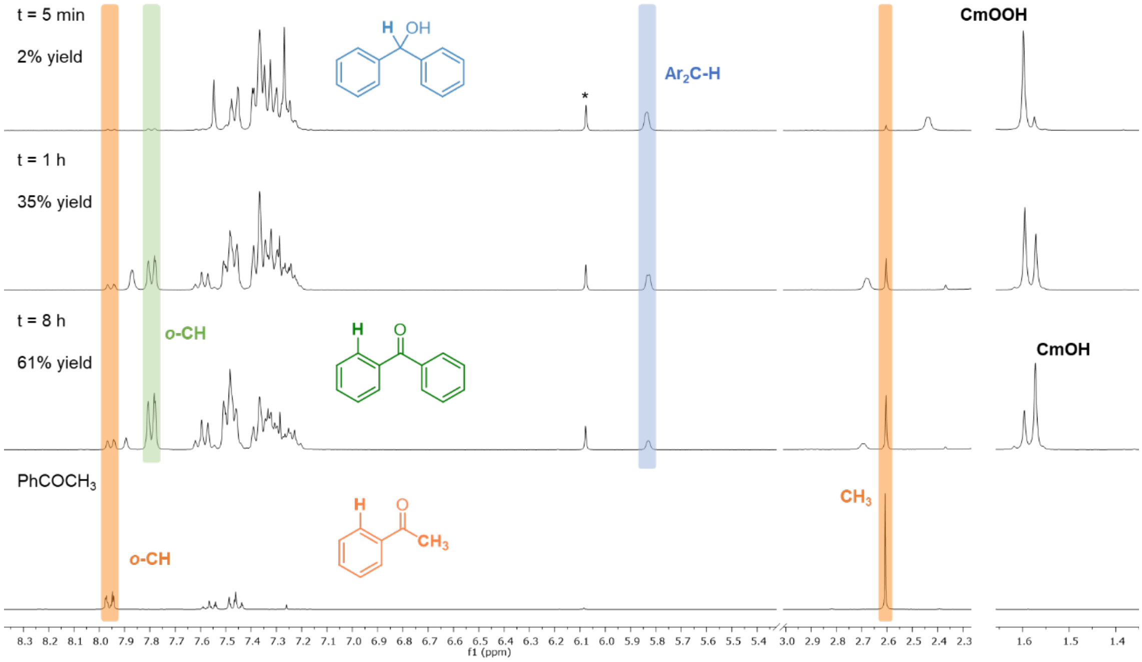

Figure S10. a) Reaction scheme of the oxidation of diphenylmethanol by cumene hydroperoxide (CmOOH) catalyzed by **2**; b) ^{1}H NMR monitoring (MeOD, 300MHz) of the oxidation reaction revealing the formation of benzophenone, cumyl alcohol, as well as acetophenone (* internal standard: 1,3,5-trimethoxybenzene). Conditions: 0.5 mmol substrate, 0.75 mmol CmOOH, 1 mol% catalyst **2**, 0.06 mmol 1,3,5-trimethoxybenzene, 0.6 mL MeCN, 60 °C, 8 h.

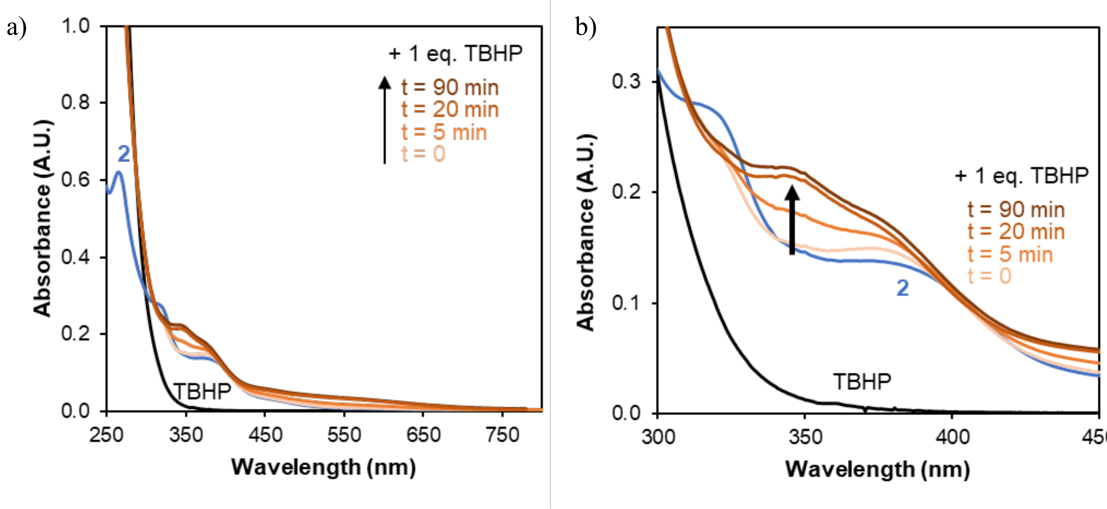

Figure S11. a) UV-vis monitoring of the reactivity of complex **2** (20 µM in MeCN) with 1 eq. TBHP; b) expansion of the 300–450 nm region.

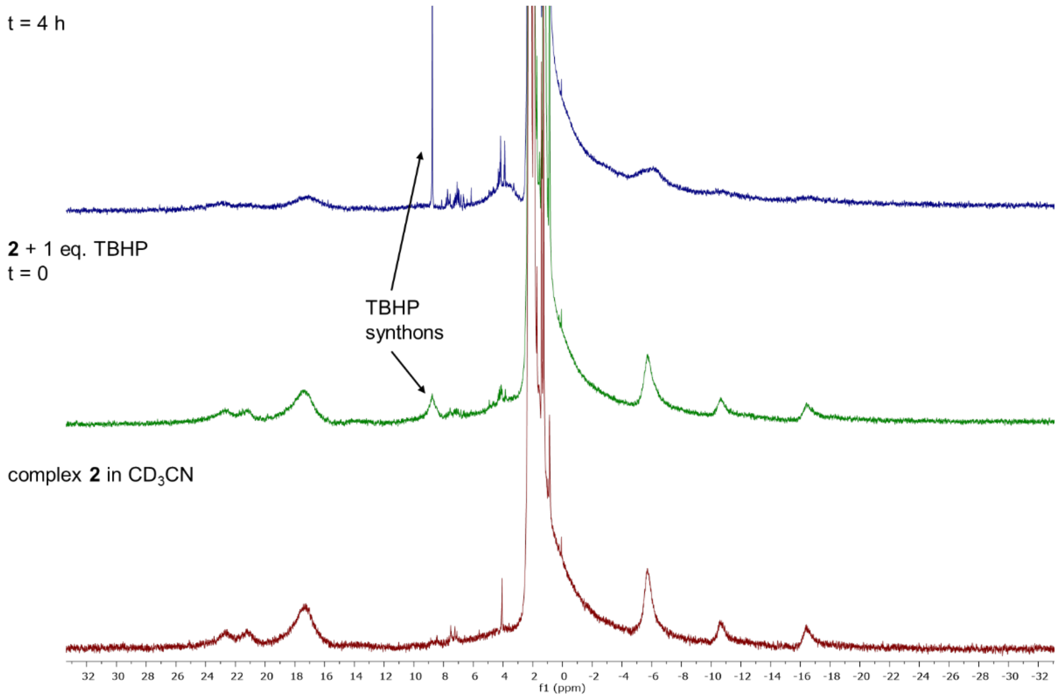

Figure S12. Monitoring of the reaction of complex **2** (10mM in CD_3CN) with 1 eq. TBHP via ^{1}H NMR spectroscopy. Bottom: spectrum of **2** before addition of TBHP; middle: spectrum immediately after addition of 1 eq. TBHP; top: spectrum after 4 h. Note that the sharp signals in the 4–10 ppm range do not correspond to those of the triazolium salt **L2** and instead were attributed to decomposition products from ligand oxidation.

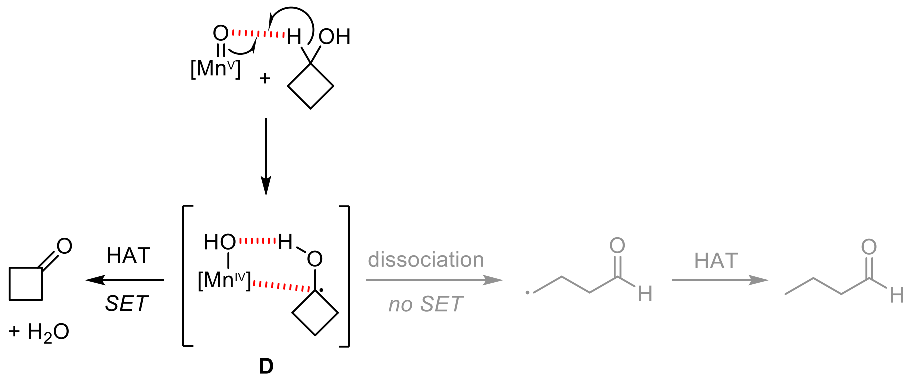

Scheme S1. Cyclobutanol oxidation schematically shown from the putative Mn-O intermediate generated upon oxidation of complex **2**: the two-electron pathway with a stable adduct **D** affords cyclobutanone, whereas the single-electron pathway due to low stability of the adduct was not observed.

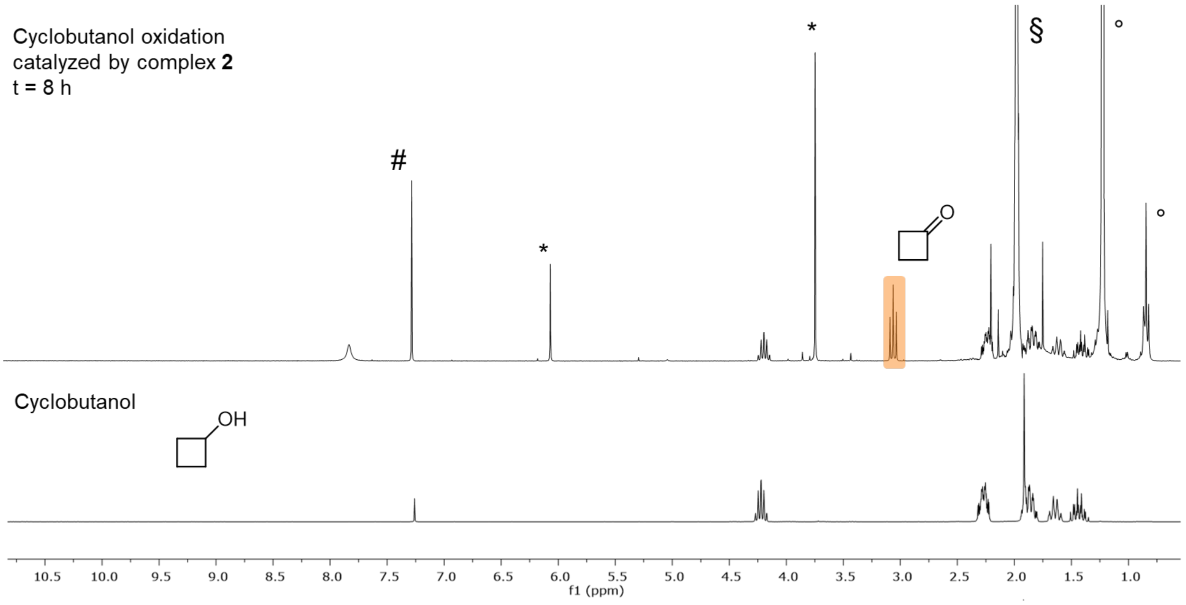

Figure S13. Monitoring of the cyclobutanol oxidation catalyzed by complex **2** via ^{1}H NMR spectroscopy (# $CHCl_3$, * internal standard: 1,3,5-trimethoxybenzene, § solvent MeCN, ° *n*-decane). Conditions: 0.5 mmol substrate, 0.75 mmol TBHP, 1 mol% catalyst **2**, 0.06 mmol 1,3,5-trimethoxybenzene, 0.6 mL MeCN, 60 °C, 8 h.

3. Characterization of the purified oxidation products

At the end of each run, the catalytic mixture was extracted with *n*-pentane (5 mL) and this phase was then dried under reduced pressure. The solid residue was purified via small-scale flash chromatography (SiO_2; CH_2Cl_2/MeOH 95:5) to afford the final oxidation product. The product yield cannot be determined by the relative integration of NMR signals of the product relative to those of the internal standard since the two species have different partition coefficients between *n*-pentane and MeCN. The spectroscopic data of the products are consistent with those reported in the literature.
Reference NMR signals of 1,3,5-trimethoxybenzene (internal standard): δ 6.08 (s, 3H, ArH), 3.76 (s, 9H, OCH_3) ppm.

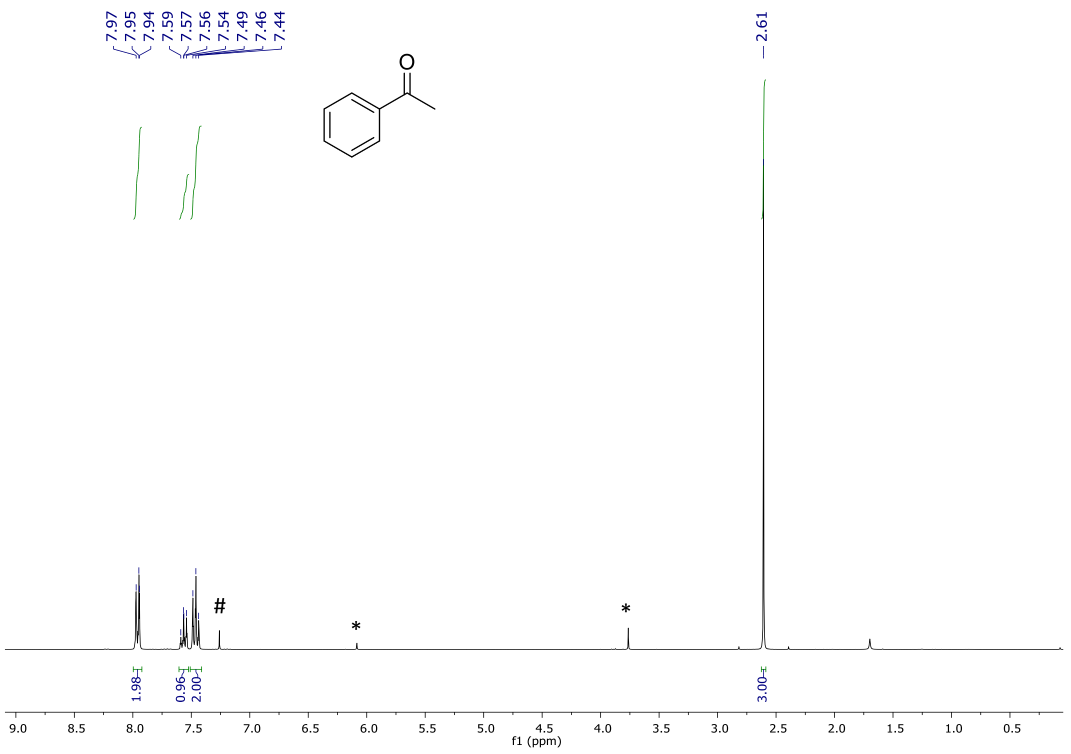

Figure S14. ^{1}H NMR spectrum ($CDCl_3$, 300MHz) of acetophenone (93% yield, from oxidation of **5a**; # $CHCl_3$, * internal standard: 1,3,5-trimethoxybenzene).

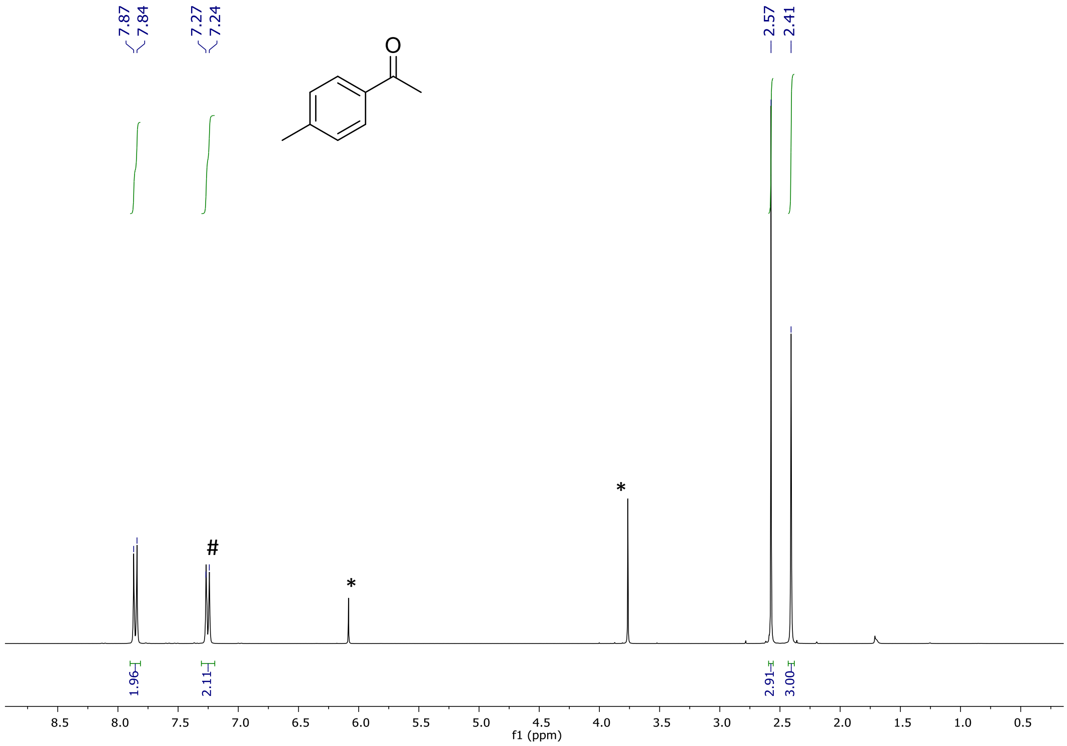

Figure S15. ^{1}H NMR spectrum ($CDCl_3$, 300MHz) of 4'-methylacetophenone (81% yield, from oxidation of **5b**; # $CHCl_3$ overlapping with a signal of the product, * internal standard: 1,3,5-trimethoxybenzene).

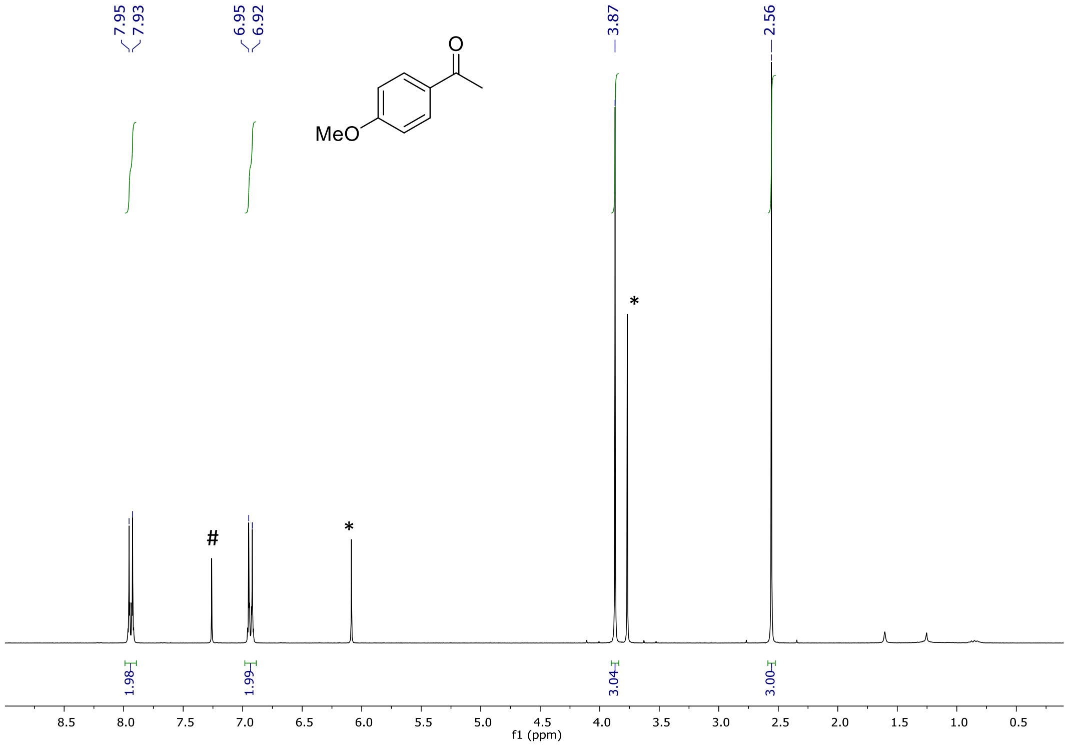

Figure S16. ^{1}H NMR spectrum ($CDCl_3$, 300MHz) of 4'-methoxyacetophenone (92% yield, from oxidation of **5c**; # $CHCl_3$, * internal standard: 1,3,5-trimethoxybenzene).

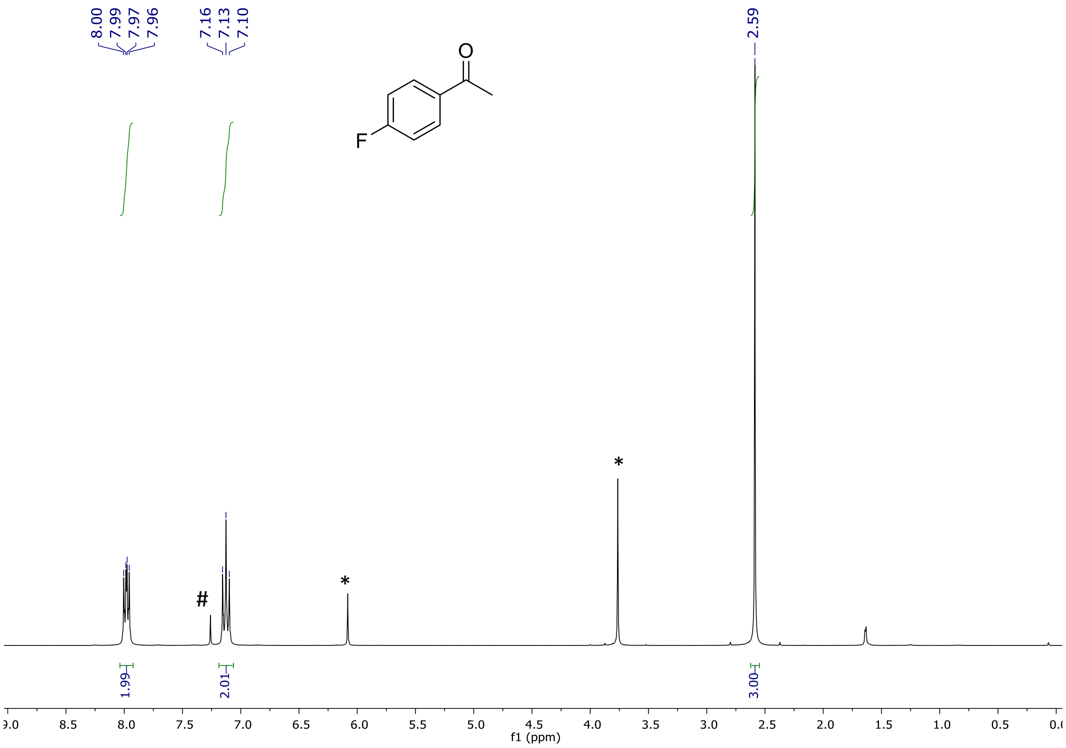

Figure S17. ^{1}H NMR spectrum ($CDCl_3$, 300MHz) of 4’-fluoroacetophenone (84% yield, from oxidation of **5d**; # $CHCl_3$, * internal standard: 1,3,5-trimethoxybenzene).

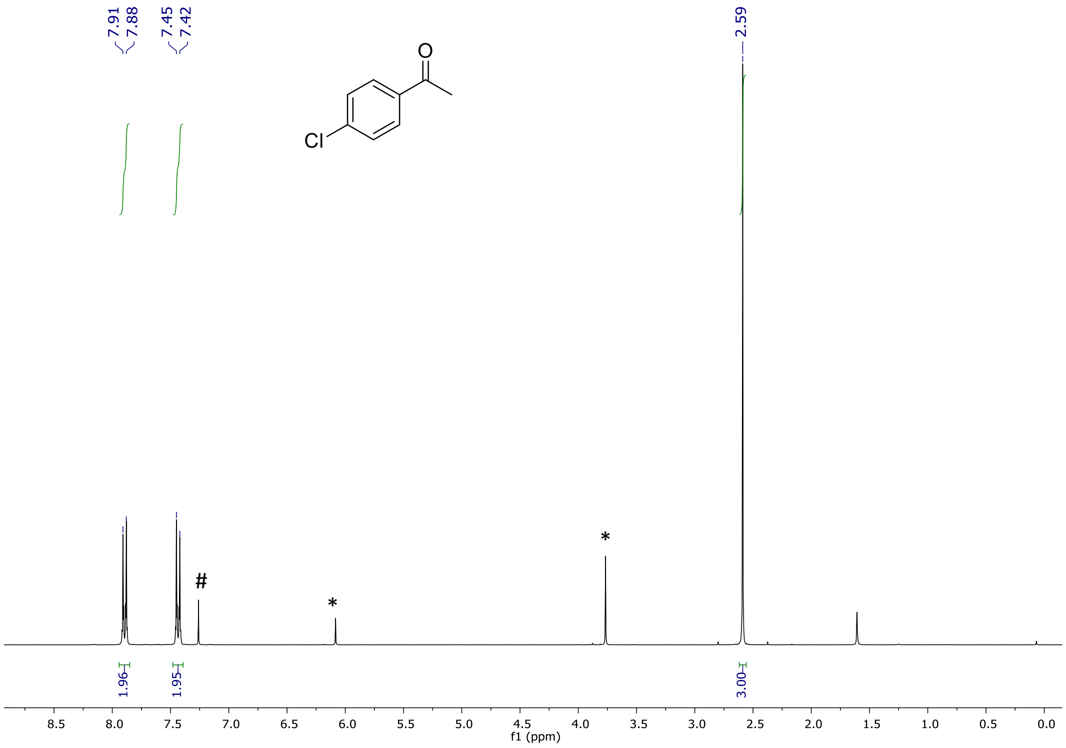

Figure S18. ^{1}H NMR spectrum ($CDCl_3$, 300MHz) of 4’-chloroacetophenone (81% yield, from oxidation of **5e**; # $CHCl_3$, * internal standard: 1,3,5-trimethoxybenzene).

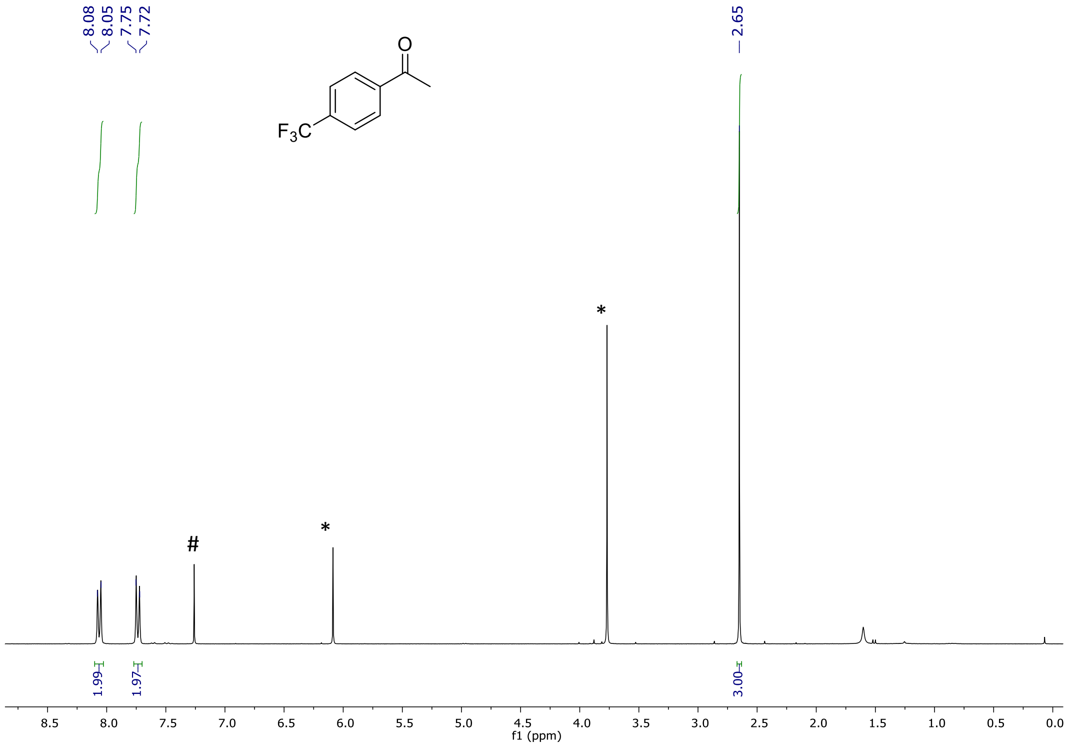

Figure S19. ^{1}H NMR spectrum ($CDCl_3$, 300MHz) of 4'-trifluoromethylacetophenone (75% yield, from oxidation of **5f**; # $CHCl_3$, * internal standard: 1,3,5-trimethoxybenzene).

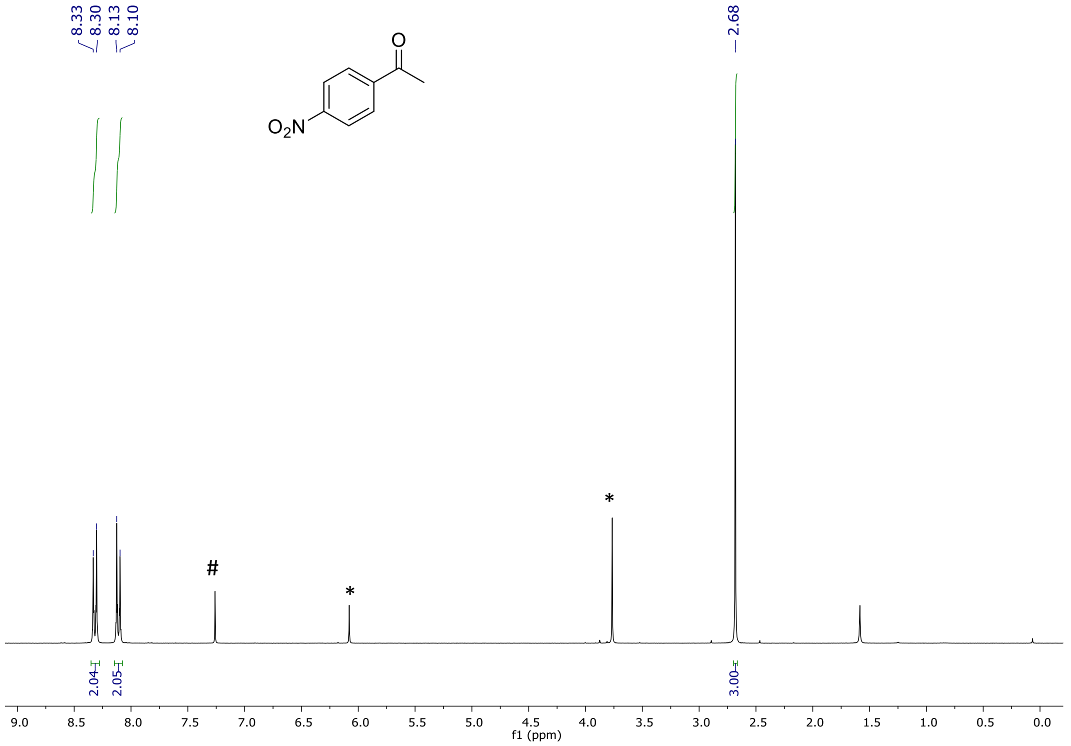

Figure S20. ^{1}H NMR spectrum ($CDCl_3$, 300MHz) of 4'-nitroacetophenone (61% yield, from oxidation of **5g**; # $CHCl_3$, * internal standard: 1,3,5-trimethoxybenzene).

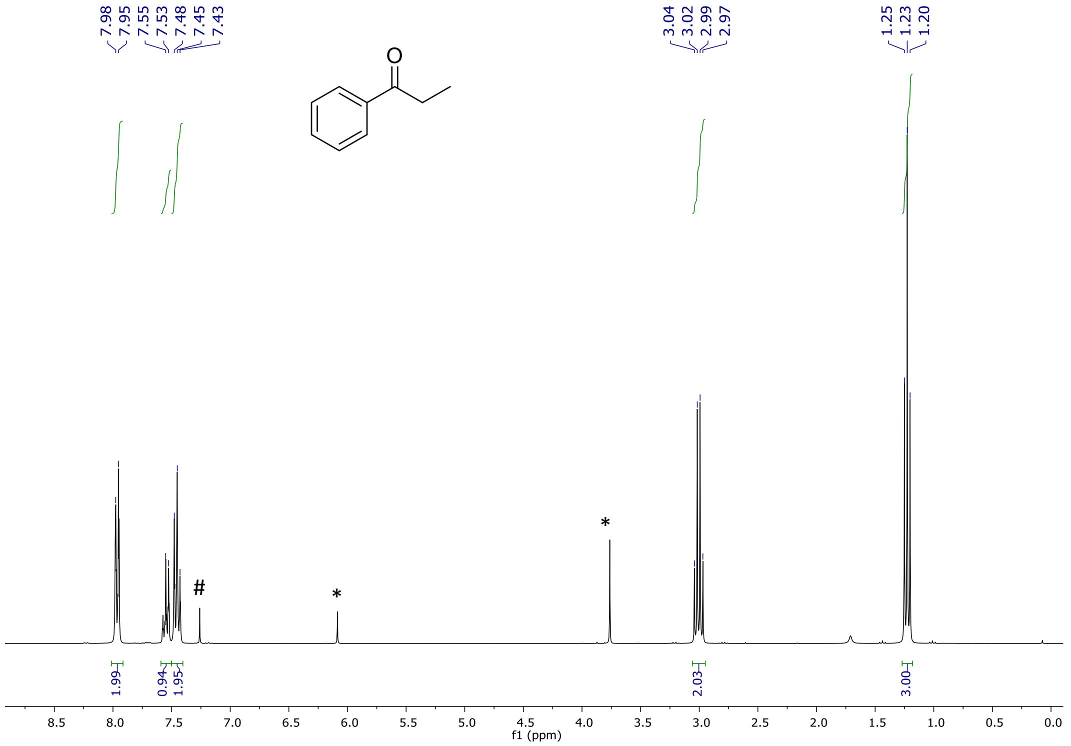

Figure S21. ^{1}H NMR spectrum ($CDCl_3$, 300MHz) of propiophenone (83% yield, from oxidation of **6**; # $CHCl_3$, * internal standard: 1,3,5-trimethoxybenzene).

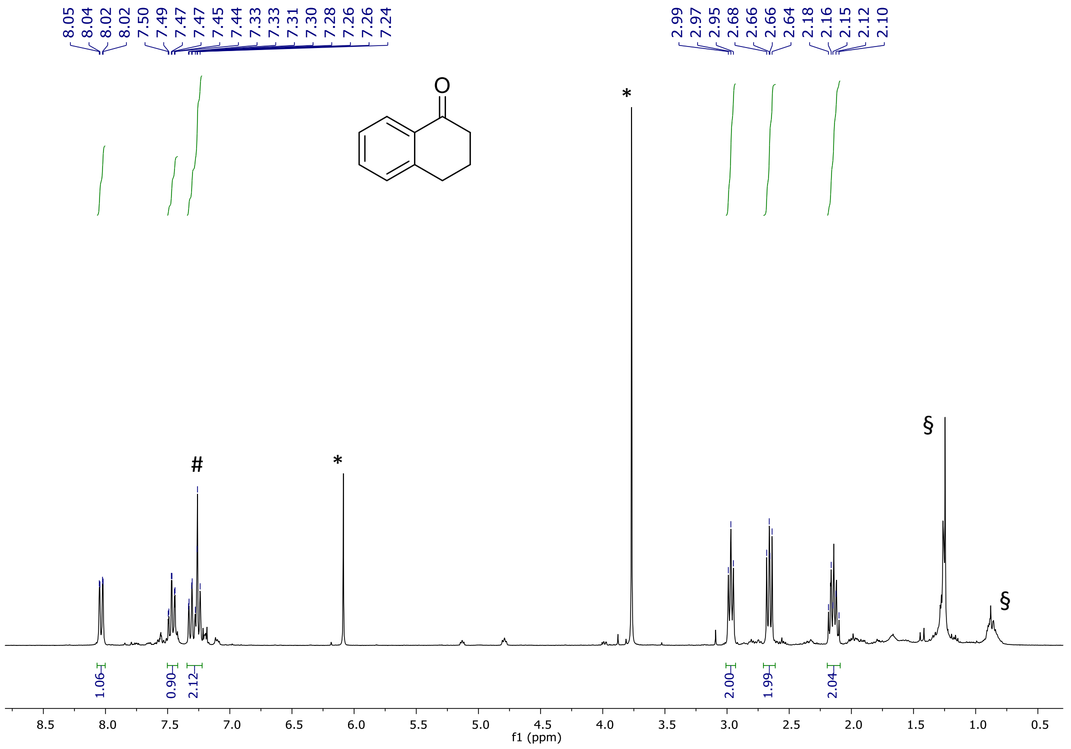

Figure S22. ^{1}H NMR spectrum ($CDCl_3$, 300MHz) of α-tetralone (81% yield, from oxidation of **7**; # $CHCl_3$ overlapping with a signal of the product, * internal standard: 1,3,5-trimethoxybenzene, § *n*-decane).

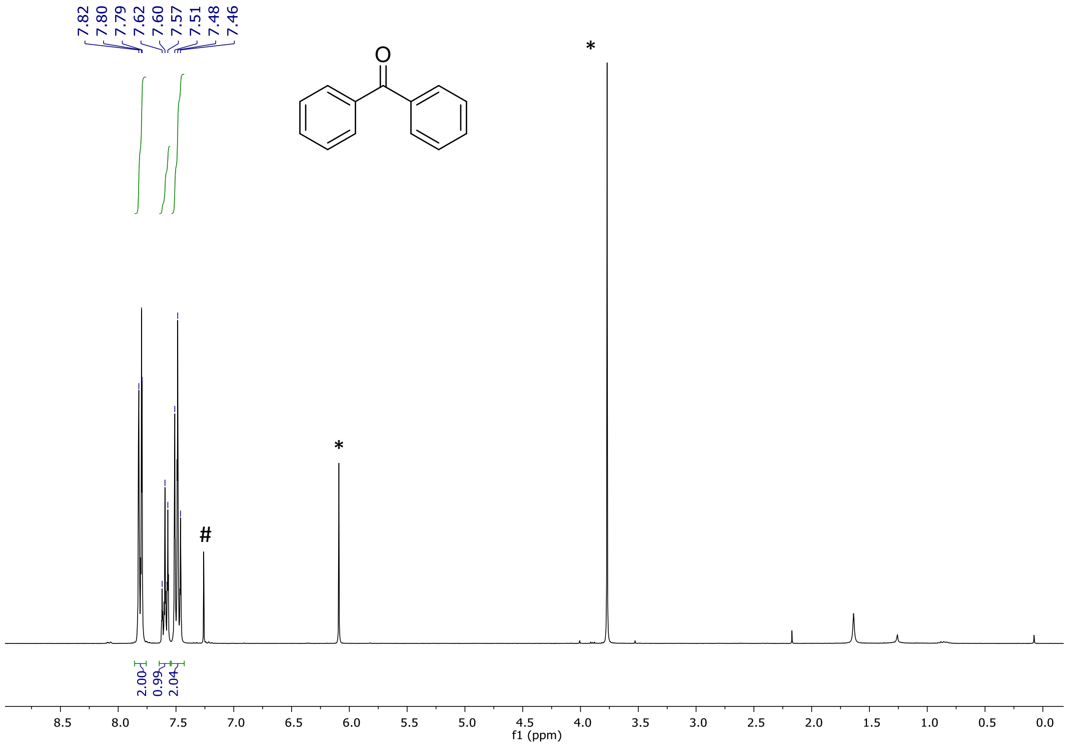

Figure S23. ^{1}H NMR spectrum ($CDCl_3$, 300MHz) of benzophenone (> 99% yield after 4 h, from oxidation of **8**; # $CHCl_3$, * internal standard: 1,3,5-trimethoxybenzene).

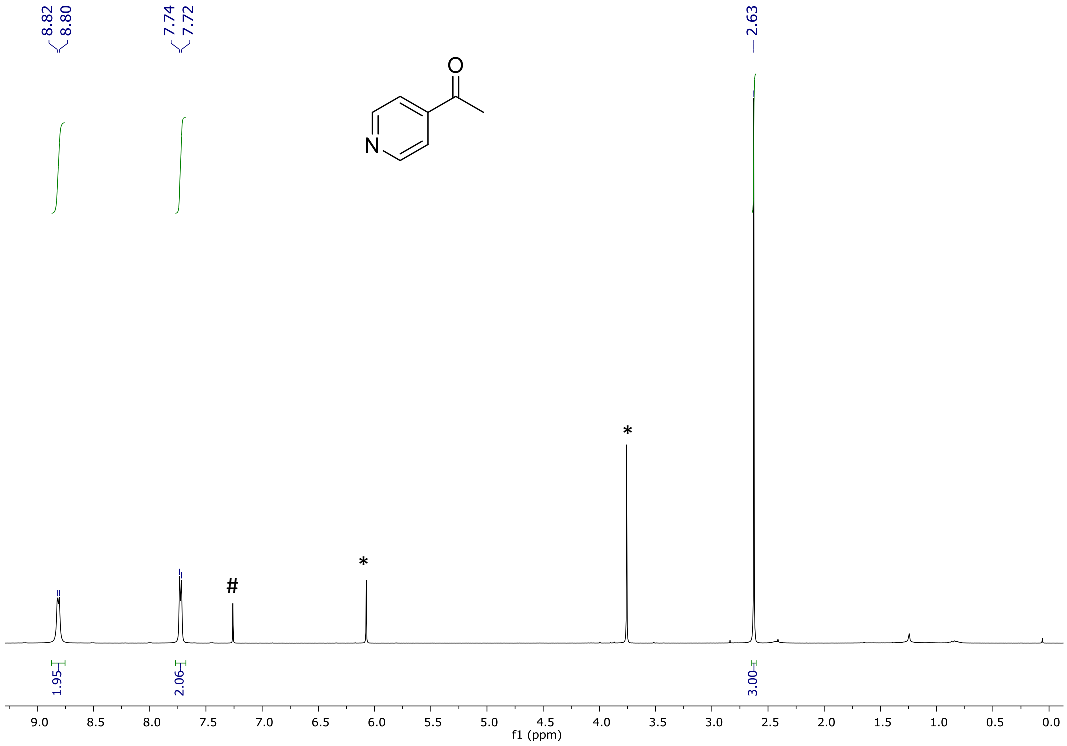

Figure S24. ^{1}H NMR spectrum ($CDCl_3$, 300MHz) of 4-acetylpyridine (> 99% yield, from oxidation of **9**; # $CHCl_3$, * internal standard: 1,3,5-trimethoxybenzene).

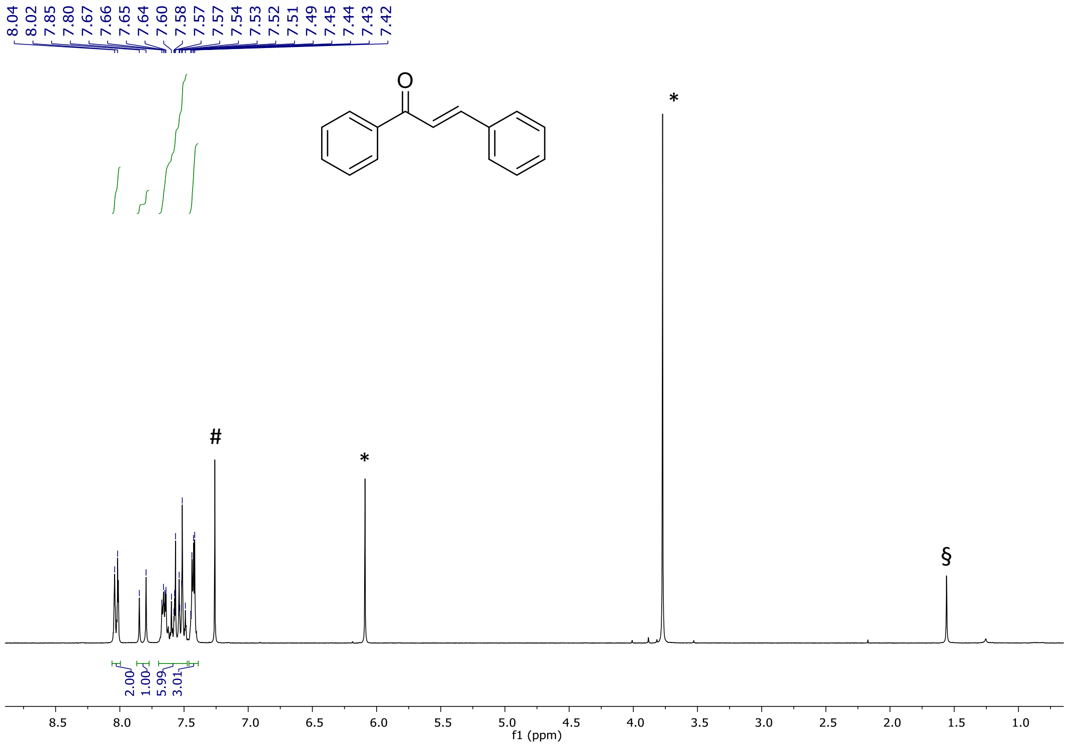

Figure S25. ^{1}H NMR spectrum ($CDCl_3$, 300MHz) of *trans*-chalcone (91% yield after 4 h, from oxidation of **10**; # $CHCl_3$, * internal standard: 1,3,5-trimethoxybenzene, § water).

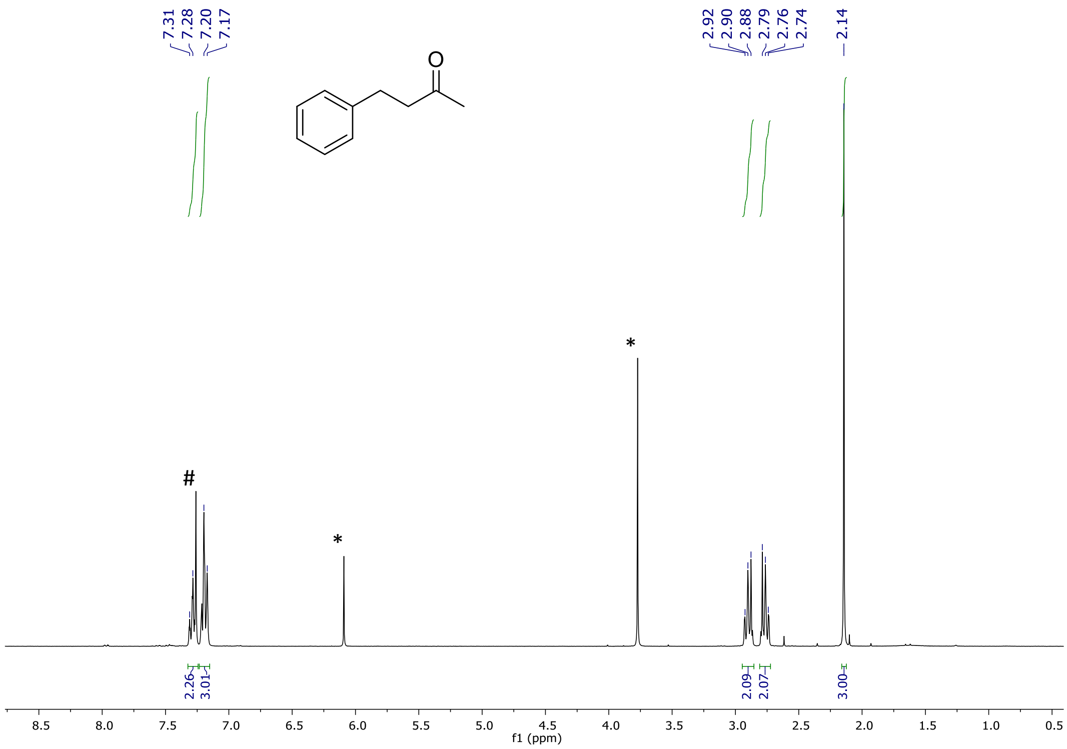

Figure S26. ^{1}H NMR spectrum ($CDCl_3$, 300MHz) of 4-phenyl-2-butanone (47% yield, from oxidation of **11**; # $CHCl_3$ overlapping with a signal of the product, * internal standard: 1,3,5-trimethoxybenzene).

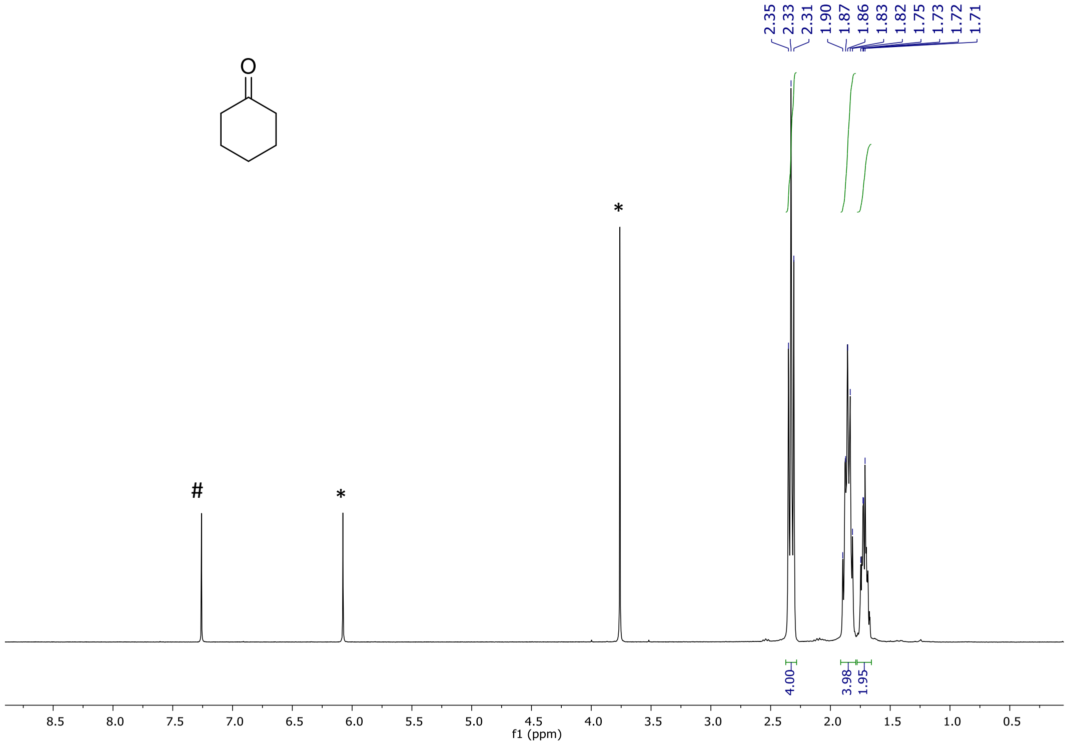

Figure S27. ¹H NMR spectrum ($CDCl_3$, 300MHz) of cyclohexanone (32% yield, from oxidation of **12**; # $CHCl_3$, * internal standard: 1,3,5-trimethoxybenzene).

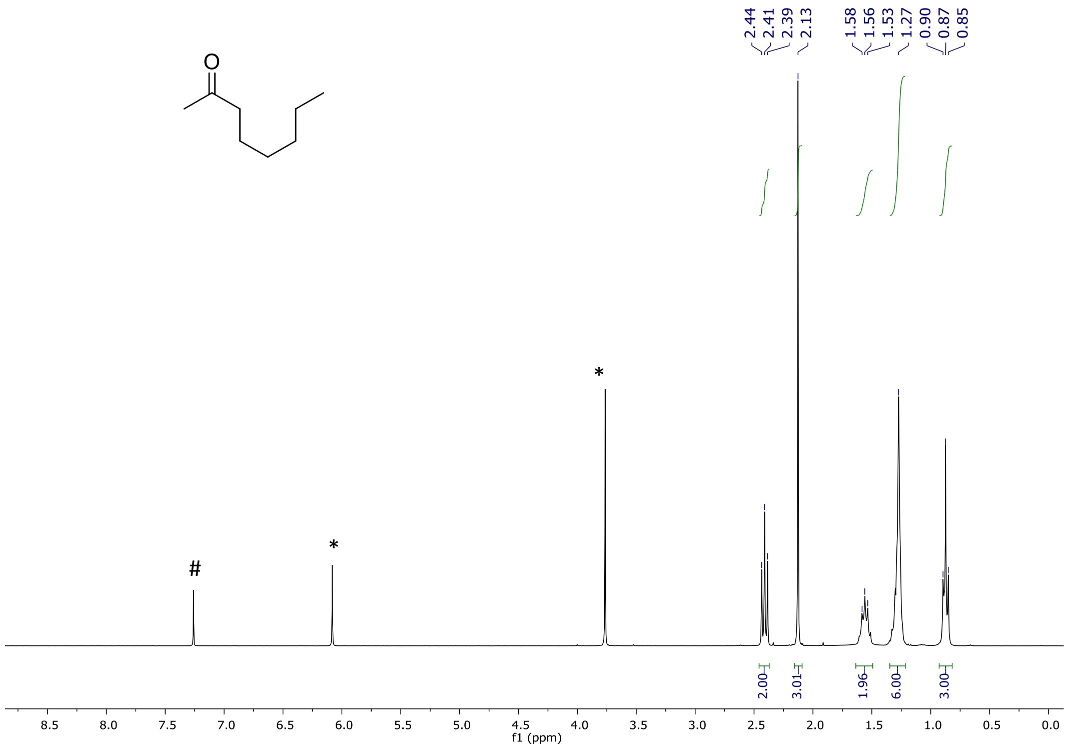

Figure S28. ¹H NMR spectrum ($CDCl_3$, 300MHz) of 2-octanone (49% yield, from oxidation of **13**; # $CHCl_3$, * internal standard: 1,3,5-trimethoxybenzene).

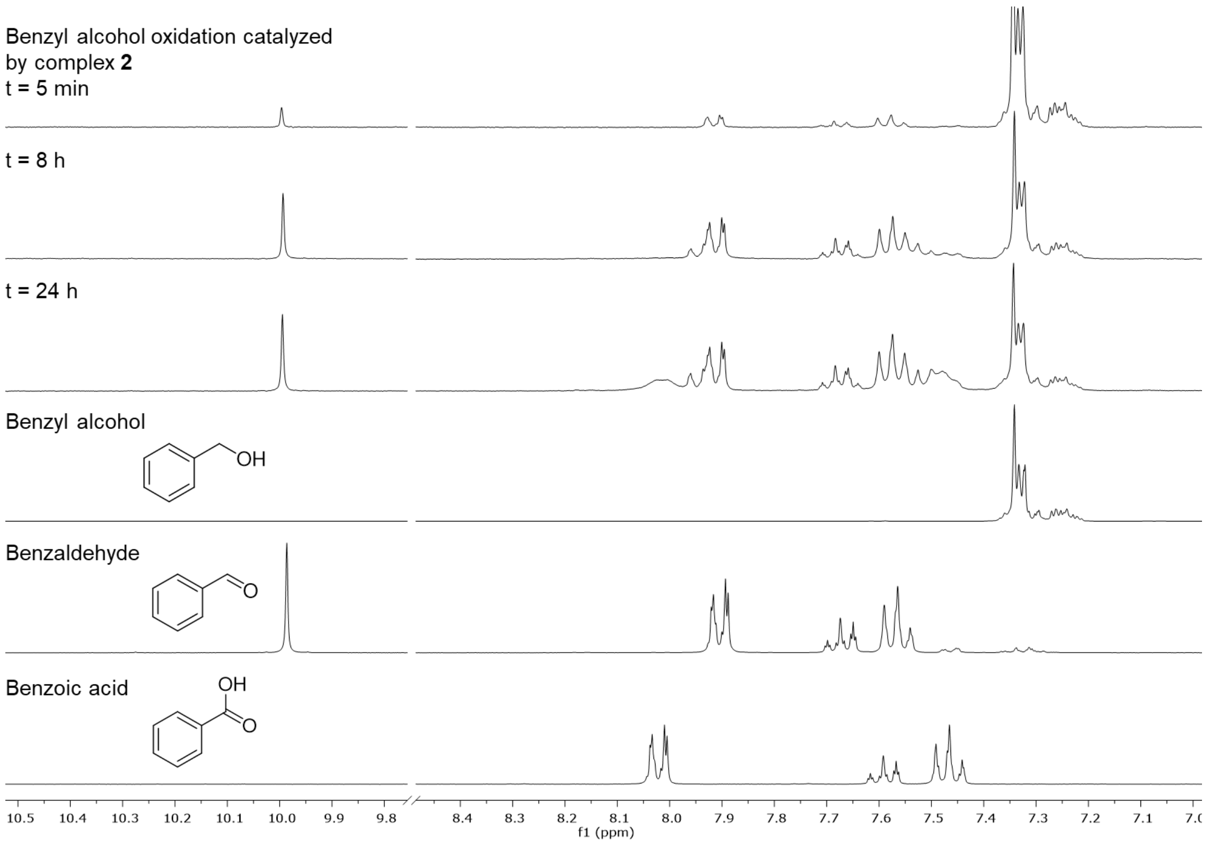

Figure S29. ^{1}H NMR monitoring (MeOD, 300MHz) of the benzyl alcohol oxidation catalyzed by complex **2**.

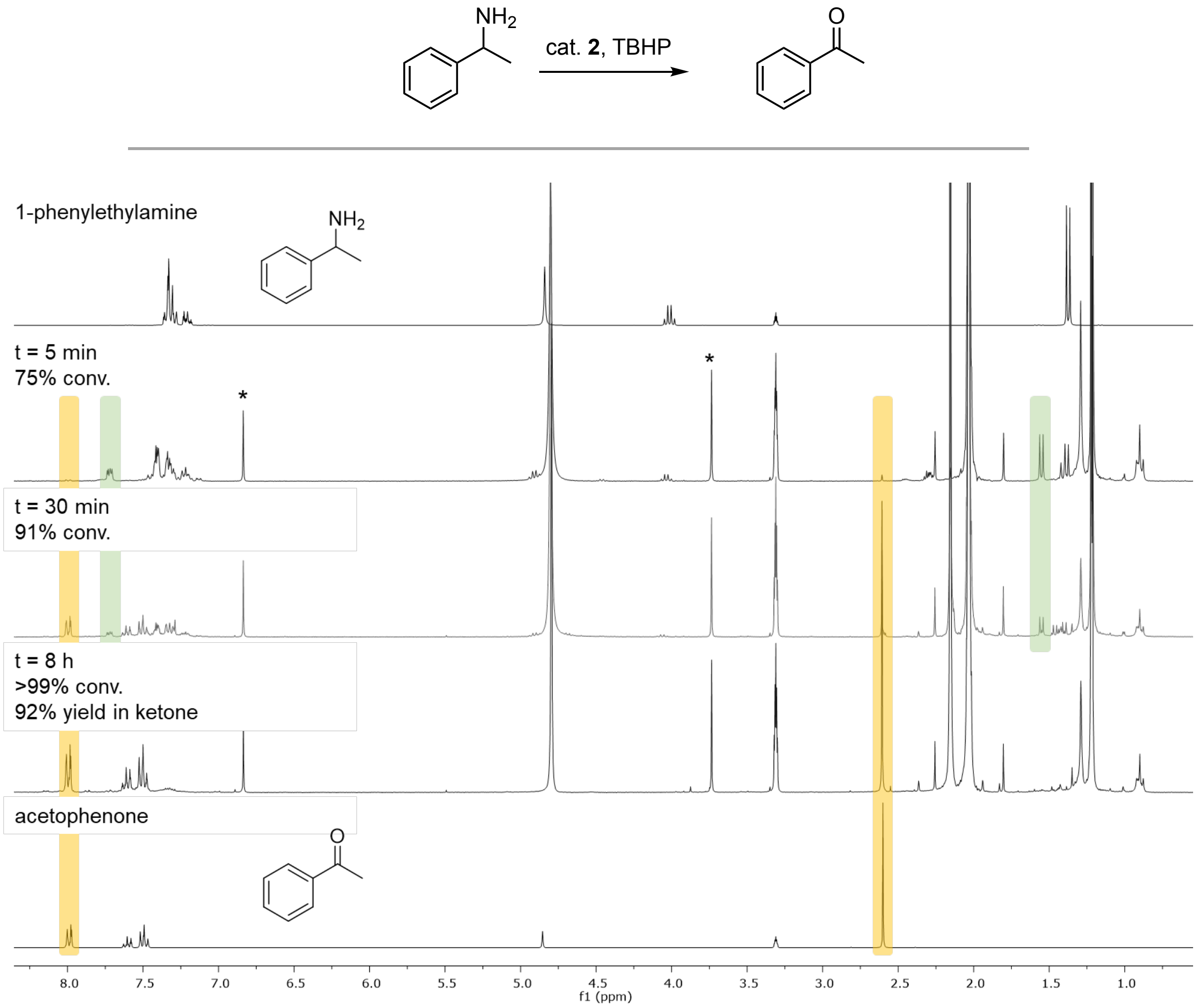

Figure S30. ^{1}H NMR spectra (MeOD, 300 MHz) from monitoring the oxidation of 1-phenylethylamine catalyzed by complex **2**, revealing the formation of a transient species (in green) and acetophenone (in yellow) (* internal standard: 1,4-dimethoxybenzene). Top and bottom spectra are reference spectra. Catalytic conditions: 0.5 mmol substrate, 0.75 mmol TBHP, 1 mol% complex **2**, 0.06 mmol 1,4-dimethoxybenzene, 0.6 mL MeCN, 60 °C, 8 h.

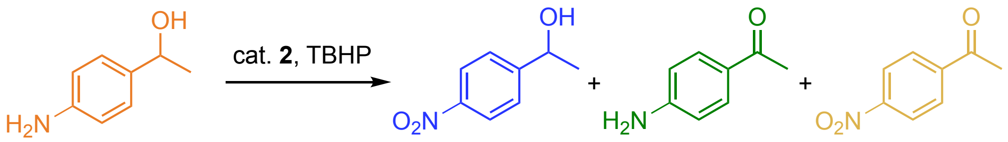

time	composition (%)			
5 min	72	22	6	< 1
30 min	6	46	13	6
4 h	< 1	42	11	8

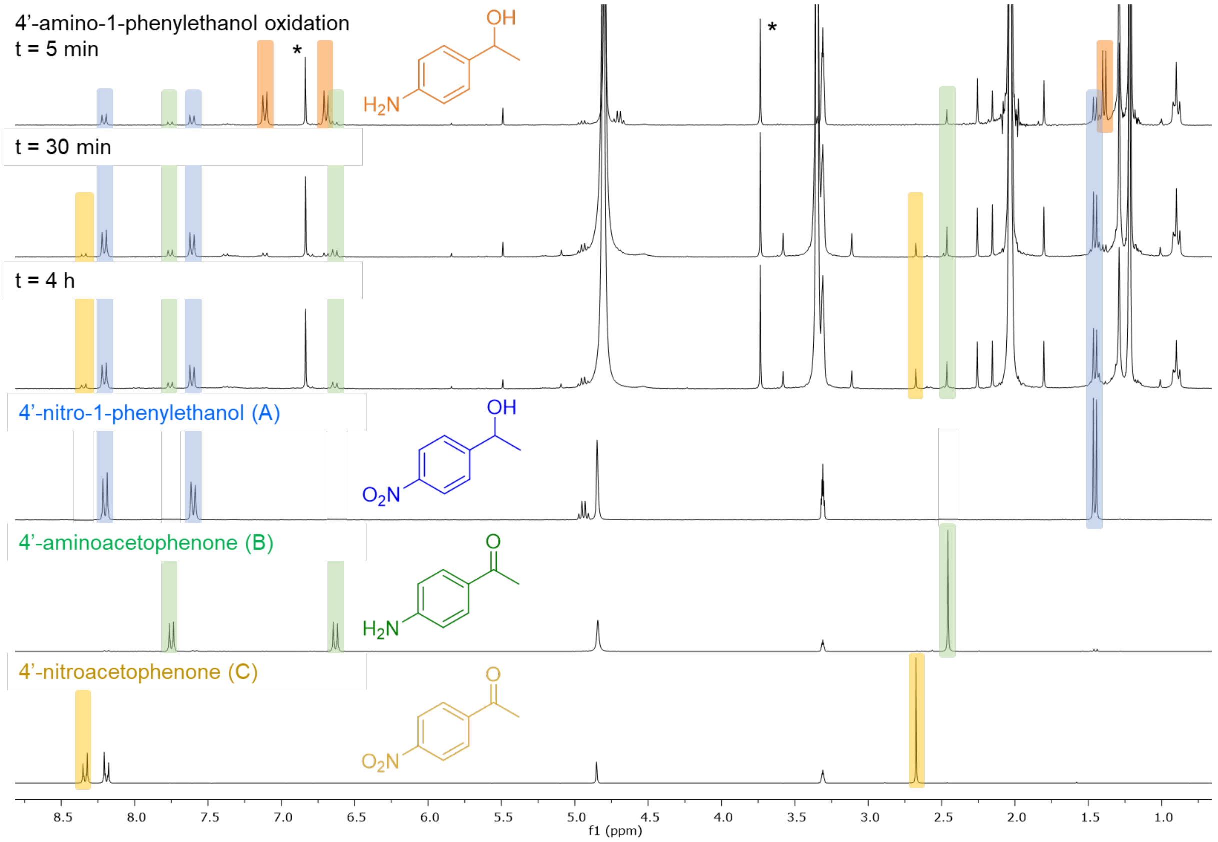

Figure S31. ^{1}H NMR spectra (MeOD, 300 MHz) from monitoring the oxidation of 4'-amino-1-phenylethanol catalyzed by complex **2**, indicating the formation of a mixture of the 4'-nitro-1-phenylethanol (in blue), 4'-aminoacetophenone (in green) and 4'-nitroacetophenone (in yellow). Lower spectra are reference compounds and diagnostic signals are highlighted, * internal standard (1,4-dimethoxybenzene). Catalytic conditions: 0.5 mmol substrate, 0.75 mmol TBHP, 1 mol% catalyst **2**, 0.06 mmol 1,4-dimethoxybenzene, 0.6 mL MeCN, 60 °C, 8 h.

4. Crystallographic details

All measurements were made on an Oxford Diffraction SuperNova area-detector diffractometer[S1] using mirror optics monochromated Mo Kα (λ = 0.71073 Å) or Cu Kα radiation (λ = 1.54184) and Al filtering.[S2] The unit cell constants and an orientation matrix for data collection were obtained from a least-squares refinement of the setting angles of reflections in the range 2.0° < θ < 27.9°. A total of 728 frames were collected using ω scans, with 8+8 seconds exposure time, a rotation angle of 1.0° per frame, a crystal-detector distance of 65.0 mm, at T = 173(2) K. Data reduction was performed using the *CrysAlisPro* program.[S1] The intensities were corrected for Lorentz and polarization effects, and a numerical absorption correction based on gaussian integration over a multifaceted crystal model was applied. Data collection and refinement parameters are presented in the Supporting Information. The structure was solved by direct methods using *SHELXT*,[S3] which revealed the positions of the non-hydrogen atoms of the title compound. All non-hydrogen atoms were refined anisotropically. All H-atoms were placed in geometrically calculated positions and refined using a riding model where each H-atom was assigned a fixed isotropic displacement parameter with a value equal to 1.2 Ueq of its parent atom (1.5 Ueq for methyl groups). Refinement of the structure was carried out on F^2 using full-matrix least squares procedures, which minimized the function $\Sigma w(F_o^2 - F_c^2)^2$. The weighting scheme was based on counting statistics and included a factor to downweight the intense reflections. All calculations were performed using the SHELXL-2014/7 program[S4] in OLEX2.[S5] Further crystallographic details are compiled in Tables S2–S4. Crystallographic data for the structures of all compounds reported in this paper have been deposited with the Cambridge Crystallographic Data Centre (CCDC) as supplementary publication numbers 2252970 (**1**), 2252969 (**2**), and 2252968 (**3**).

Table S2. Crystal data and structure refinement for **1**.

CCDC No	2252970
Empirical formula	$C_{36}H_{49}MnN_2O_4$
Formula weight	628.71
Temperature/K	173.00(10)
Crystal system	triclinic
Space group	P-1
a/Å	10.2841(3)
b/Å	13.6750(5)
c/Å	14.2509(5)
α/°	62.126(4)
β/°	80.257(3)
γ/°	78.894(3)
Volume/Å^3	1731.22(12)
Z	2
ρ_{calc}/gcm^{-3}	1.206
F(000)	672.0
Crystal size/mm^3	0.252 × 0.184 × 0.133
Radiation	Mo Kα (λ = 0.71073)
2Θ range for data collection/°	3.246 to 52.728
Reflections collected	40807
Independent reflections	7068 [R_{int} = 0.0555, R_{sigma} = 0.0385]
Data/restraints/parameters	7068/0/402
Goodness-of-fit on F^2	1.077

Final R indexes [I>=2σ (I)]	R_1 = 0.0751, wR_2 = 0.2050
Final R indexes [all data]	R_1 = 0.0878, wR_2 = 0.2158
Largest diff. peak/hole /eA^{-3}	2.56/-0.39

Table S3. Crystal data and structure refinement for **2**.

CCDC No	2252969
Empirical formula	$C_{36}H_{50}MnN_3O_4$
Formula weight	643.73
Temperature/K	173.00(10)
Crystal system	monoclinic
Space group	$P2_{1/c}$
a/A	16.41996(13)
b/A	11.69669(11)
c/A	18.63705(16)
α/°	90
β/°	98.1747(8)
γ/°	90
Volume/A^3	3543.05(5)
Z	4
ρ_{calc}/gcm^{-3}	1.207
μ/mm^{-1}	0.413
F(000)	1376.0
Crystal size/mm^3	0.295 × 0.267 × 0.234
Radiation	Mo Kα (λ = 0.71073)
2Θ range for data collection/°	4.124 to 56.308
Reflections collected	30799
Independent reflections	7983 [R_{int} = 0.0286, R_{sigma} = 0.0276]
Data/restraints/parameters	7983/0/412
Goodness-of-fit on F^2	1.042
Final R indexes [I>=2σ (I)]	R_1 = 0.0353, wR_2 = 0.0879
Final R indexes [all data]	R_1 = 0.0457, wR_2 = 0.0951
Largest diff. peak/hole /eA^{-3}	0.27/-0.30

Table S4. Crystal data and structure refinement for **3**.

CCDC No	2252968
Empirical formula	$C_{44}H_{40}Mn_2N_6O_8$
Formula weight	890.70
Temperature/K	234(80)
Crystal system	triclinic
Space group	P-1
a/A	9.8533(2)
b/A	10.0379(2)
c/A	10.7928(3)
α/°	88.126(2)
β/°	76.940(2)
γ/°	74.520(2)
Volume/A^3	1001.71(4)

Z	1
ρ_{calc}/gcm^{-3}	1.477
μ/mm^{-1}	5.656
F(000)	460.0
Crystal size/mm^3	0.142 × 0.078 × 0.066
Radiation	Cu Kα (λ = 1.54184)
2Θ range for data collection/°	8.414 to 127.372
Reflections collected	12749
Independent reflections	3210 [R_{int} = 0.0291, R_{sigma} = 0.0252]
Data/restraints/parameters	3210/0/274
Goodness-of-fit on F^2	1.060
Final R indexes [I>=2σ (I)]	R_1 = 0.0266, wR_2 = 0.0719
Final R indexes [all data]	R_1 = 0.0301, wR_2 = 0.0735
Largest diff. peak/hole /eA^{-3}	0.20/-0.18

5. References

S1 Oxford Diffraction Ltd., *CrysAlisPro* (Version 1.171.40.39a); Oxford Diffraction Ltd., Yarnton, Oxfordshire, UK, 2018, pp. 279−288..

S2 P. Macchi, H.-B. Bürgi, A. S. Chimpri, J. Hauser and Z. Gál, *J. Appl. Crystallogr.*, 2011, **44**, 763–771.

S3 G. M. Sheldrick, *Acta Crystallogr. Sect. A: Found. Adv.*, 2015, **71**, 3–8.

S4 G. M. Sheldrick, *Acta Crystallogr. Sect. C: Struct. Chem.*, 2015, **71**, 3–8.

S5 O. V. Dolomanov, L. J. Bourhis, R. J. Gildea, J. A. K. Howard and H. Puschmann, *J. Appl. Crystallogr.*, 2009, **42**, 339–341.
